# Late Miocene transformation of Mediterranean Sea biodiversity

**DOI:** 10.1126/sciadv.adp1134

**Published:** 2024-09-25

**Authors:** Konstantina Agiadi, Niklas Hohmann, Elsa Gliozzi, Danae Thivaiou, Francesca R. Bosellini, Marco Taviani, Giovanni Bianucci, Alberto Collareta, Laurent Londeix, Costanza Faranda, Francesca Bulian, Efterpi Koskeridou, Francesca Lozar, Alan Maria Mancini, Stefano Dominici, Pierre Moissette, Ildefonso Bajo Campos, Enrico Borghi, George Iliopoulos, Assimina Antonarakou, George Kontakiotis, Evangelia Besiou, Stergios D. Zarkogiannis, Mathias Harzhauser, Francisco Javier Sierro, Marta Coll, Iuliana Vasiliev, Angelo Camerlenghi, Daniel García-Castellanos

**Affiliations:** ^1^ Department of Geology, University of Vienna, Josef-Holaubek-Platz 2, Geozentrum, 1090 Vienna, Austria.; ^2^ Faculty of Geosciences, Department of Earth Sciences, Utrecht University, Vening Meineszgebouw A, Princetonlaan 8a, 3584 CB Utrecht, Netherlands.; ^3^ Institute of Evolutionary Biology, University of Warsaw, Krakowskie Przedmieście 26/28, 00-927 Warsaw, Poland.; ^4^ Dipartimento di Scienze, Università Roma Tre, L.go S. Leonardo Murialdo, 1, 00146 Roma, Italy.; ^5^ Natural History Museum of Basel, Augustinergasse 2, 4001 Basel, Switzerland.; ^6^ Department of Historical Geology and Palaeontology, Faculty of Geology and Geoenvironment, National and Kapodistrian University of Athens, Panepistimiopolis Zografou, 15784 Athens, Greece.; ^7^ Dipartimento di Scienze Chimiche e Geologiche, Università degli Studi di Modena e Reggio Emilia, 09124 Cagliari, Italy.; ^8^ Institute of Marine Science - National Research Council, ISMAR-CNR, Via Gobetti 101, 40129 Bologna, Italy.; ^9^ Stazione Zoologica ‘Anton Dohrn’, Villa Comunale, Via Caracciolo, 80122 Napoli, Italy.; ^10^ Dipartimento di Scienze della Terra, Università di Pisa, 56126 Pisa, Italy.; ^11^ Université de Bordeaux/UMR ‘EPOC’ CNRS 5805, allée Geoffroy St-Hilaire, 33615 Pessac Cedex, France.; ^12^ Department of Geology, University of Salamanca, Plaza de Los Caidos s/n, 37008 Salamanca, Spain.; ^13^ Groningen Institute of Archaeology, University of Groningen, Postsraat 6, 9712 Groningen, Netherlands.; ^14^ Department of Earth Sciences, University of Torino, Via Valperga Caluso 35, 10125 Torino, Italy.; ^15^ Museo di Storia Naturale, Università degli Studi di Firenze, 50121 Florence, Italy.; ^16^ Sección de Paleontología, Museo de Alcalá de Guadaíra, 41500 Seville, Spain.; ^17^ Società Reggiana di Scienze Naturali, 42122 Reggio Emilia, Italy.; ^18^ Department of Geology, University of Patras, University Campus, 26504 Rio, Achaia, Greece.; ^19^ Department of Earth Sciences, University of Oxford, OXI 3AN Oxford, UK.; ^20^ Natural History Museum, Burgring 7, 1010 Vienna, Austria.; ^21^ Institute of Marine Science (ICM-CSIC), Passeig Marítim de la Barceloneta 37-49, 08003 Barcelona, Spain.; ^22^ Senckenberg Biodiversity and Climate Research Centre (BiK-F), Georg-Voigt-Straße 14-16, 60325 Frankfurt am Main, Germany.; ^23^ OGS Istituto Nazionale di Oceanografia e di Geofisica Sperimentale, 34010 Trieste, Italy.; ^24^ Geosciences Barcelona (GEO3BCN-CSIC), Solé i Sabarís s/n, 08028, Barcelona, Spain.

## Abstract

Understanding deep-time marine biodiversity change under the combined effects of climate and connectivity changes is fundamental for predicting the impacts of modern climate change in semi-enclosed seas. We quantify the Late Miocene–Early Pliocene [11.63 to 3.6 million years (Ma)] taxonomic diversity of the Mediterranean Sea for calcareous nannoplankton, dinocysts, foraminifera, ostracods, corals, molluscs, bryozoans, echinoids, fishes, and marine mammals. During this time, marine biota was affected by global climate cooling and the restriction of the Mediterranean’s connection to the Atlantic Ocean that peaked with the Messinian salinity crisis. Although the net change in species richness from the Tortonian to the Zanclean varies by group, species turnover is greater than 30% in all cases, reflecting a high degree of reorganization of the marine ecosystem after the crisis. The results show a clear perturbation already in the pre-evaporitic Messinian (7.25 to 5.97 Ma), with patterns differing among groups and subbasins.

## Teaser

Global climate cooling, basin restriction, and the Messinian salinity crisis transformed Mediterranean biodiversity.

## INTRODUCTION

Climate and connectivity control the structure and functioning of marine ecosystems (*1*). Although this statement is supported by theory and observation, the response of the different groups of organisms to combined climate-connectivity changes remains unclear. The Mediterranean is a model marginal oceanic basin, whose ecosystem is profoundly altered by climate and connectivity changes today. A semi-enclosed basin, the Mediterranean is, at present, one of the places most affected by climate warming (*2*), as well as by impressive rates of invasion by alien species from the Indo-Pacific realm after the opening of the Suez Canal in 1869 (*3*) and potentially from the tropical Atlantic in the near future (*4*). Under such dynamic conditions, it is challenging to predict community and ecosystem future states, and it is therefore important to look to the geological past for periods of extreme environmental change. In this respect, the Late Miocene Mediterranean Sea is the ideal setting.

**Fig. 1.**
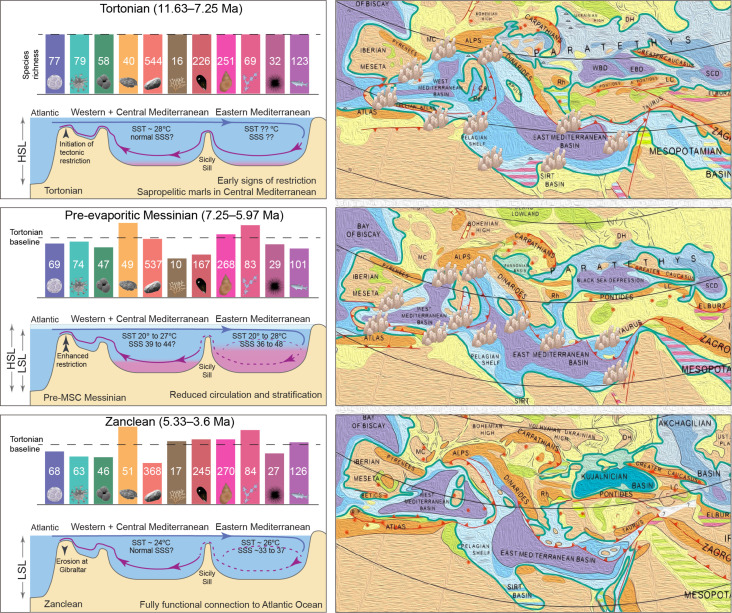
Species richness for each group of organisms, paleogeography, and paleoceanographic conditions across the Mediterranean basin during the Tortonian, pre-evaporitic Messinian, and Zanclean. The Messinian salinity crisis (MSC) led to a restructuring of the marine biota in the Mediterranean, as shown by the divergent contributions of each group to the post-MSC biota compared to the Tortonian levels. The colors and icons on each bar correspond to those in Fig. 2. Richness values are the medians obtained by our analysis using random subsampling at 80% for the three intervals (full results shown in Fig. 2). The species richness bars are normalized to the Tortonian levels, which are considered the baseline here. Paleogeographic maps are modified on the basis of (*92*). The locations on the maps of the coral reefs have been approximated on the basis of the occurrences of *Porites* corals in the revised dataset (*35*); *Porites* coral reefs were extensive before the MSC but completely vanished afterward (*93*, *94*). Sea surface temperature (SST) and salinity (SSS) values come from (*8*, *13*, *15*, *95*, *96*). HSL, high sea level; LSL, low sea level.

The Late Miocene [11.63 to 5.33 million years (Ma)] was a pivotal time for the Mediterranean marine biota. The Mediterranean, once part of the Western Tethys tropical biodiversity hot spot (*5*, *6*), formed as a distinct sea, separated from the Indian Ocean after the closure of the Tethys Seaway ~13.8 Ma (*7*). In the Late Miocene, the global climate cooling (*8*) and the basin’s stepwise restriction from the Atlantic Ocean preceding the Messinian salinity crisis (MSC; Fig. 1) (*9*–*12*) led to extreme sensitivity to climatic perturbations within the Mediterranean, which manifested as high-amplitude variability in both temperature and salinity (Fig. 1) (*13*–*16*). Yet, the impact of the resulting ecological crisis on marine biodiversity has never been systematically studied.

High-stress conditions for marine organisms have been reported from the earliest Messinian (7.17 Ma) (*17*, *18*). Gradually, the restriction of the Rifian and Betic corridors (in present-day North Morocco and South Spain, respectively; Fig. 1) led to strong water-column stratification and episodes of dysoxia on the sea bottom (Fig. 1) (*13*, *19*). The first MSC evaporites were deposited on the Mediterranean marginal basins at 5.97 Ma, although Zachariasse and Lourens (*20*) suggested that the MSC started already at 6 Ma, at least in the Eastern Mediterranean. There is general consensus that a one-way connection between the Mediterranean and the Atlantic was maintained at least during the initial stage of the MSC (5.97 to 5.6 Ma) (*12*, *14*, *21*). Even more extreme conditions occurred during the second MSC stage (5.6 to 5.55 Ma) (*22*), as evidenced by the kilometer-thick deposits of salt found across the deeper parts of the Mediterranean. In the final stage of the MSC, periodic alternations of gypsum and marls (5.55 to 5.42 Ma) followed by the brackish “Lago Mare” deposits (5.42 to 5.33 Ma) reflect an increase of freshwater influx to the basin possibly from the Paratethys in the North (*23*), although the Paratethyan-inflow hypothesis has been contested (*24*). Normal marine conditions were established once more in the Mediterranean at the base of the Zanclean at 5.33 Ma (*25*), after the restoration of the connection with the Atlantic Ocean (Fig. 1) (*26*).

The literature is full of hypotheses on the magnitude of the MSC repercussions on the different groups of organisms [e.g., (*27*)], but these are mostly based on incomplete and still uncertain scenarios about the MSC, as well as on the intuitive assumption that such a paleoenvironmental perturbation must have caused a “major” change in the marine biota. Having prevailed for many decades now, this assumption has leaked from paleontology and geosciences to biological sciences, with numerous papers referring to it as a fact [e.g., (*28*)], instead of what it truly is, an assumption. Pioneering studies that investigated effects of the MSC on specific taxonomic groups [e.g., (*29*, *30*)] are in need of revision, as the stratigraphic placement and taxonomic identification of the fossils have often been revised since their publication. In contrast, some studies have supported an opposite view: For example, Néraudeau *et al.* (*31*) stated that the “Messinian desiccation was not a drastic event for irregular echinoids”, and Goubert *et al.* (*32*), studying the benthic foraminifera assemblages at Los Yesos (Sorbas Basin, Spain), supported that “the MSC is not associated with a biological crisis.” Moreover, Monegatti and Raffi (*33*) reported an important impact of the MSC on marine gastropods, but a very small impact on bivalves, highlighting the need for a more in-depth, integrated ecosystem–based assessment.

In this study, we analyze a recently revised Tortonian-Zanclean marine fossil record of calcareous nannoplankton, dinocysts, planktic and benthic foraminifera, ostracods, corals, bivalves, gastropods, bryozoans, echinoids, bony fishes, sharks, and marine mammals from the Western Mediterranean, the Eastern Mediterranean, and the Po Plain–Northern Adriatic region (figs. S1 and S2) (*34*, *35*) to obtain evidence of changes in the taxonomic diversity of the Mediterranean marine biota that took place from the time of the initiation of the Mediterranean-Atlantic gateway restriction in the late Tortonian (*36*), until the reestablishment of a fully marine environment in the Zanclean. Investigations of the fossil record from the MSC beds have been presented elsewhere, indicating that stenohaline marine organisms appeared in various levels (*37*, *38*). In our investigation, we exclude the MSC interval because these fossil records are very limited compared to the records before and after the crisis and insufficient for the present biodiversity analysis. Instead, we take a step back and evaluate the biodiversity change by comparing the Tortonian to the pre-evaporitic Messinian and to the Zanclean fossil record of these groups. Taxonomic diversity is examined by calculating four diversity metrics: richness, total dissimilarity (Sørensen index), dissimilarity due to turnover (Simpson index), and nestedness (*39*). Our results quantify and demonstrate the impact that changes in basin connectivity and climate had on the composition of the marine assemblages of the different groups and highlight research questions that remain open.

## RESULTS

**Fig. 2.**
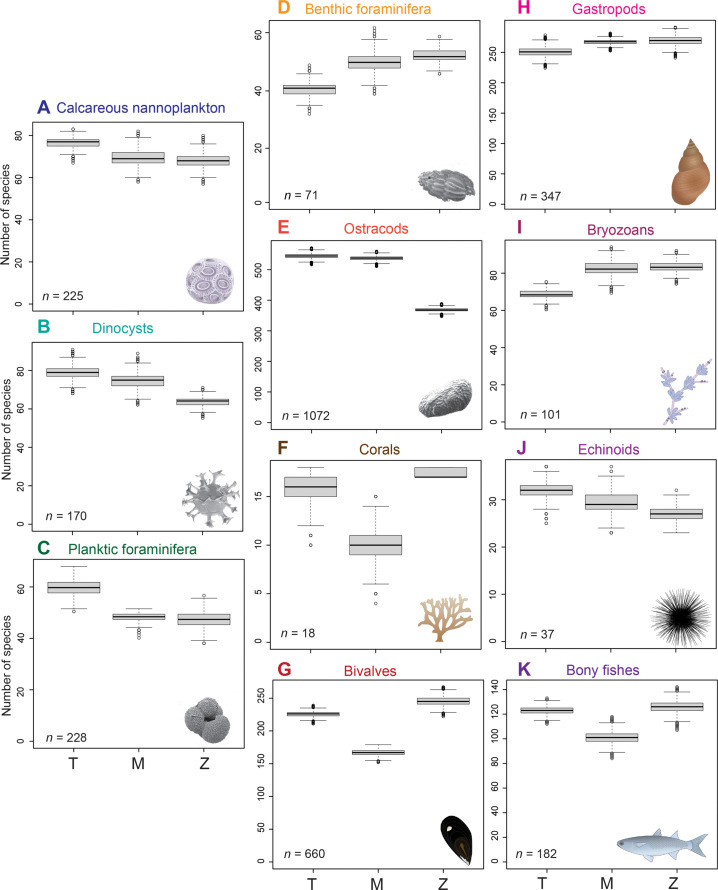
Changes in species richness of the Mediterranean Sea biota from the Late Miocene to the Early Pliocene by group of organisms. (**A** to **K**) The *x* axes shows intervals: Tortonian (T), pre-evaporitic Messinian (M), and Zanclean (Z). The species richness of corals in the Zanclean is only due to azooxanthellate corals because zooxanthellate (reef-building) corals are not present in the Mediterranean after the MSC. Symbols for organisms obtained from the Integration and Application Network (ian.umces.edu/media-library). *n* indicates the number of occurrences after subsampling to 80% of the smallest sample, 10,000 times. The bold line indicates the median value, the box corresponds to the quartiles (values included fall within 25th and 75th percentiles of the data), and whiskers are quartiles plus/minus 1.5 times the interquartile range.

The Late Miocene–Early Pliocene Mediterranean species richness patterns vary greatly between taxonomic groups (Figs. 1 and 2) and subbasins (Supplementary Materials). Species richness decreased from the Tortonian to the pre-evaporitic Messinian for calcareous nannoplankton (by 11.6%; driven by the Western Mediterranean; fig. S4), dinocysts (5.3%; also driven by the Western Mediterranean; fig. S5), planktic foraminifera (23.4%; mostly in the Western Mediterranean; fig. S6), corals [60%; although *n* = 18; this is driven by zooxanthellate reef-building corals (z-corals); fig. S9], bivalves (35.3%; large decrease in the Western Mediterranean but increase in the Eastern Mediterranean; fig. S8), echinoids (10.3%; driven by the Western Mediterranean, where most data come from; fig. S2), and bony fishes (21.8%; driven by Po Plain–Northern Adriatic region; fig. S11) (two-sample one-tailed Wilcoxon test on rarefied gradient, *P* < 2 × 10^−16^). In contrast, ostracod species richness slightly increased from the Tortonian to the Messinian, but this is driven by the Po Plain–Northern Adriatic record, while richness increased more in both the Western and Eastern Mediterranean (fig. S7). Species richness increased also from the Tortonian to the Messinian for benthic foraminifera (18.4%), gastropods (6.3%; driven by the Po Plain–Northern Adriatic), and bryozoans (16.9%; fig. S10) (one-sided one-sample Wilcoxon test, *P* < 10^−10^). From the Messinian to the Zanclean, species richness slightly increased for calcareous nannoplankton (increases in the Western but decreases in the Eastern Mediterranean; fig. S4) but slightly decreased for planktic and benthic foraminifera, gastropods, and bryozoans. There is a more considerable decrease in richness for dinocysts (19.1%; driven by the Eastern Mediterranean; fig. S5), ostracods (45.9%; also mostly driven by the Eastern Mediterranean; fig. S7), and echinoids (7.4%) (one-sided one-sample Wilcoxon test, *P* < 10^−10^). Species richness increased from the Messinian to the Zanclean for corals [41.2%; only cold, deep-water corals in the Zanclean; fig. S9; (*33*)], bivalves (32.1%), and bony fishes (19.8%). Reef-building z-corals are absent in the Mediterranean after the MSC, and, therefore, their species richness in the Zanclean basically derives from azooxanthellate (cold, deep-water) corals (az-corals; fig. S9).

Species (Fig. 2) and genus (fig. S3) richness show the same patterns, with few exceptions. Genus richness of calcareous nannoplankton increases from the pre-evaporitic Messinian to the Zanclean (fig. S3A) driven by the Western Mediterranean (fig. S4), while species richness continues to decrease (Fig. 2A) driven by the Eastern Mediterranean (fig. S4). For echinoids, genus richness increases (fig. S3J), but species richness decreases (Fig. 2J) from the Tortonian to the pre-evaporitic Messinian (in both cases, one-sample one-tail Wilcoxon test, *P* < 2 × 10^−6^).

**Fig. 3.**
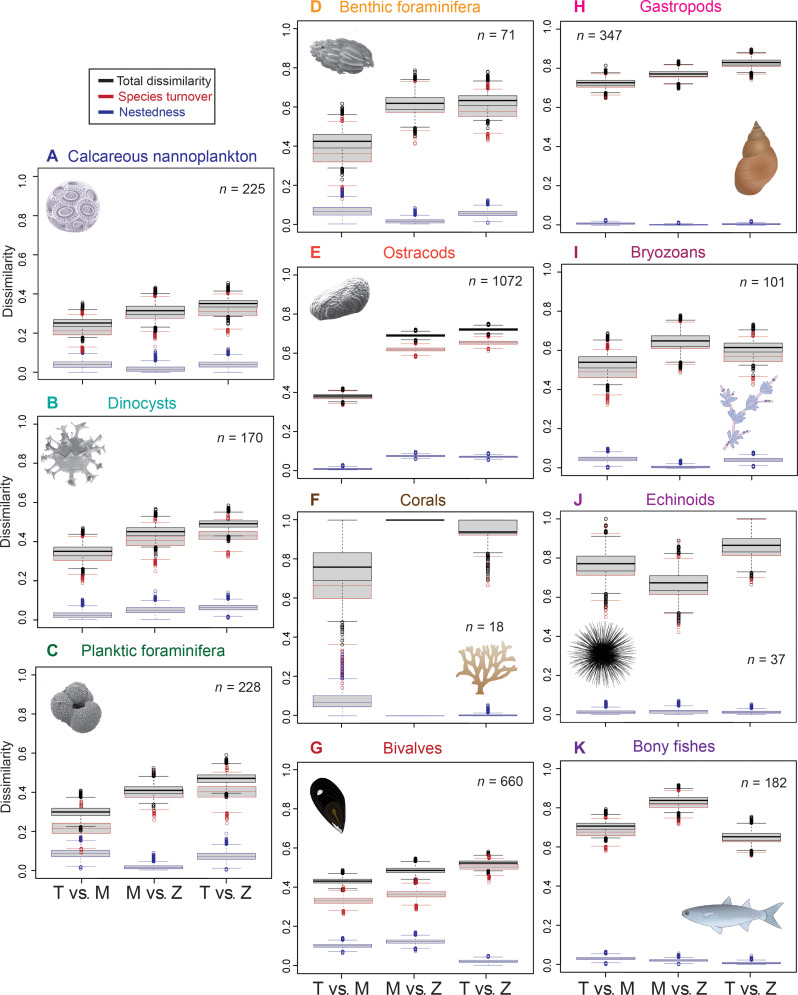
Temporal change: Dissimilarities between the Tortonian (T), pre-evaporitic Messinian (M), and Zanclean (Z) biodiversities of the Mediterranean for each group of organisms. (**A** to **K**) Black, total dissimilarity; red, species turnover; blue, nestedness.

Species turnover always contributes more than nestedness to the total dissimilarity, in all comparisons (Fig. 3). Total dissimilarity and species turnover are higher in the Messinian-versus-Zanclean comparisons of all groups, whereas nestedness is highest in the Tortonian–versus–pre-evaporitic Messinian comparisons. Total dissimilarity between the Tortonian and pre-evaporitic Messinian records exceeds 50% for corals (76.0%), gastropods (72.8%; driven by the Po Plain–Northern Adriatic), bryozoans (53.9%), echinoids (77.4%), and bony fishes (70.6%), whereas it is less than 50% for calcareous nannoplankton (25.2%), dinocysts (35.0%), planktic (30.0%), and benthic foraminifera (42.2%), ostracods (37.8%), and bivalves (43.0%). In the Messinian-versus-Zanclean comparisons, total dissimilarity is greater than 50% in benthic foraminifera (61.6%), ostracods (68.7%), corals (100%), gastropods (77.2%), bryozoans (64.9%), echinoids (67.3%), and bony fishes (83.7%). Only calcareous nannoplankton (31.4%), dinocysts (45.5%), planktic foraminifera (40.7%), and bivalves (48.4%) maintain lower than 50% dissimilarities.

Last, the net effect (Tortonian-versus-Zanclean comparisons) varies among groups, with species turnover exceeding 30% in all groups: 31.0% for calcareous nannoplankton, 41.7% for dinocysts, 40.0% for planktic and 57.5% for benthic foraminifera, 65.0% for ostracods, and 93.8% corals. These values of species turnover are at the levels achieved during the pre-evaporitic Messinian. Gastropods and echinoids exhibit even higher species turnover in the Tortonian-versus-Zanclean (82.5 and 85.2%) than in the Messinian-versus Zanclean comparison (77.0 and 65.4%, respectively). The Tortonian-versus-Zanclean faunas of bryozoans (61.5%) and bony fishes (65.2%) are more similar than the Messinian-versus-Zanclean faunas (64.7 and 83.7%, respectively).

## DISCUSSION

### Climatic and connectivity impacts

The Late Miocene–Early Pliocene Mediterranean marine biota resulted from the interplay between global climatic cooling and changes in marine connectivity within and beyond the Mediterranean Sea (Fig. 1 and Table 1).

**Table 1.** The main observed biodiversity changes in the different groups of marine organisms in the Mediterranean Sea during the Late Miocene and beginning of the Pliocene.

Major group	Taxonomic group	Main biodiversity changes
Plankton	Calcareous nannoplankton	Drop in species richness first in the Western Mediterranean already from pre-evaporitic Messinian attributed to eutrophication due to gateway restriction and then in the Eastern subbasin after the MSC
	Dinoflagellate cysts	
	Planktic foraminifera	Species richness decrease already in the pre-evaporitic Messinian in both subbasins, probably due to the high-amplitude salinity fluctuations
Benthos	Ostracods	Assemblages (particularly bathyal ones) less abundant and less diversified in the Messinian; drop in species richness in the Zanclean, mostly in the Eastern Mediterranean
	Bivalves	Drop in species richness in the pre-evaporitic Messinian and recovery in the Zanclean
	Echinoids	Drop in species richness in the pre-evaporitic Messinian due to the salinity and temperature fluctuations but further decrease in the Zanclean
	Azooxanthellate corals	Increase in species richness in the pre-evaporitic Messinian, but assemblages contain species adapted to low oxygen- and high-organic matter content
	Gastropods	
	Bryozoans	
	Benthic foraminifera	
Coral reefs	Zooxanthellate corals	Already negatively affected by Late Miocene cooling; decrease in species richness in the pre-evaporitic Messinian: by the early Messinian, reefs in the Mediterranean were built almost exclusively by Porites; complete extirpation of reef corals by the Zanclean
Nekton	Bony fishes	Drop in species richness in the pre-evaporitic Messinian and recovery in the Zanclean
Large marine vertebrates	Sharks	Follow global patterns; first appearance of the great white shark, the blue shark, and oceanic dolphins in the Mediterranean (and for the sharks globally) around the Miocene/Pliocene boundary
	Marine mammals	

#### Coral reef biodiversity

The cooling directly affected temperature-sensitive organisms such as the tropical reef-building z-corals and their associated faunas (reef fishes and sharks) and bryozoans, leading to local extinction of vast populations, particularly in the Eastern Mediterranean (Fig. 1) (*40*). In addition, the decrease of water temperatures in the Mediterranean allowed boreal species to expand their distribution to the basin during the Messinian, while strongly thermophilic Tethyan relic species disappeared. Monegatti and Raffi (*33*) noted that the MSC caused regional mass disappearances of molluscs but only a limited number of actual extinctions and that the greatest Messinian extinctions took place in the Atlantic Ocean and were triggered by the TG22, TG20, TG14, and TG12 glacials during the MSC. In the Zanclean, the establishment of psychrospheric water masses in the Atlantic further exacerbated this impact (*41*). For example, the great white shark (*Carcharodon carcharias*) and the blue shark (*Prionace glauca*) first appeared globally at the Miocene/Pliocene boundary (*42*) and in the Mediterranean after the MSC (*43*).

The MSC played a crucial role in the local extinction of shallow-water z-coral reefs, but it was probably not the main driver (*40*, *44*). Z-corals, as tropical reef corals, are highly sensitive to temperature. The distribution of coral reefs had started to shift globally, well before the MSC, from the Eocene Tethyan tropical biodiversity hot spot to the present-day Indo-Pacific hot spot (*5*, *6*, *45*). The marked decrease in their diversity within the Mediterranean is attributed to the gradual northward shift of the region outside the tropical belt due to global cooling (*44*) and the closure of the seaway with the Indo-Pacific in the late Middle Miocene (*7*, *46*). Shallow-water tropical coral reefs dominated by colonial z-corals exhibited lower diversity already in the Tortonian, which was reduced even further in the Messinian (Fig. 2F), and they disappeared from the Mediterranean after the MSC (Fig. 1) (*47*). The high species turnover between the pre- and post-MSC coral faunas (Fig. 3F) and the apparent full recovery of the species richness of corals as a whole (Fig. 2F) are due to the fact that the Pliocene Mediterranean corals were mostly deep-water azooxanthellate species (fig. S9).

#### Marine refuges during the MSC

It is unclear whether climate or the water-column stratification and increased bottom-water salinity in the Messinian drove populations to seek refuge in the Eastern Atlantic off the western coast of Africa. The Pliocene survival of Mediterranean endemics taking refuge in the Atlantic during the MSC and then repopulating the Mediterranean after the crisis has been postulated by many researchers (*48*). Considering that the overall species turnover between Tortonian and Zanclean ostracod faunas is greater than that between Tortonian and Messinian (Fig. 3E), it seems likely that the Zanclean saw a return of some ostracod species that were present in the Mediterranean during the Tortonian but not the Messinian. Within the Mediterranean, in the pre-evaporitic Messinian, the restricted conditions and high-amplitude temperature and salinity variations are reflected in the marine ostracod assemblages, which became less abundant and less diversified (*49*). The Atlantic side of the Rif cordillera (Rharb Basin, Morocco) and the Guadalquivir Basin and Gulf of Cadiz (southern Portugal and southwestern Spain) may have been such Messinian refuges (*33*). It has been suggested that some echinoid species found refuge in the North Atlantic and then repopulated the basin at the beginning of the Pliocene [e.g., (*31*)], but such a hypothesis is not supported by the fossil records of the Eastern Atlantic [e.g., (*50*)] that show similar taxonomic composition but lower diversity than their coeval communities in the Mediterranean basin. A scenario where the West Alboran Basin was a refuge for Mediterranean endemic species (*51*) is also rejected because the West Alboran Basin was disconnected from that of the Atlantic already by 7.17 Ma (*18*, *52*). Moreover, migrations to Eastern Atlantic refuges, although possible for a few species of dinocysts, ostracods, bryozoans, bivalves, gastropods, corals, and fishes, are not supported by the nestedness between the pre- and post-MSC faunas, which is consistently below 10% (Fig. 3). Instead, this nestedness (which is relatively low compared to species turnover) is better explained by the reestablishment of species that were already present in the Atlantic before the MSC.

#### Impacts on plankton

The marine gateway restriction in the pre-MSC Messinian created intervals of salinity fluctuations, bottom-water deoxygenation, and stratification of the water column (*13*, *15*), conditions that gradually became too extreme for the species inhabiting the basin during the Messinian. Most planktonic groups show a clear response, their diversity dropping in the pre-evaporitic Messinian (Fig. 2). For example, calcareous nannoplankton, dinocysts, and planktic foraminifera decreased in species richness in the Western Mediterranean during the Tortonian/Messinian transition (figs. S4 to S6), and this has been attributed to eutrophication as a result of the gateways’ restriction (*18*, *53*). In contrast, calcareous nannoplankton species richness remains quite unchanged in the Eastern Mediterranean during the pre-evaporitic Messinian (fig. S4), which has been attributed to a higher nutrient input provided through continental runoff in this region (*54*), leading to a slightly more diverse assemblage already in the Tortonian, and so the effect of the restriction was not as strong there. On the other hand, the intra-Messinian salinity fluctuations strongly affected plankton biodiversity in the Eastern Mediterranean (*55*).

#### Impacts on benthos

Regarding benthos, eutrophication and bottom-water deoxygenation negatively affected species richness during the early Messinian (*17*, *18*), but the change depicted in the deep bottom environments is not captured in the overall record (Fig. 2) because of the appearance of shallow-water species (*55*). Particularly, littoral ostracod faunas were not at all impoverished, but bathyal ostracod assemblages were affected by the gateway restriction and the changes in water circulation (*56*). Deep-water coral communities (both colonial and solitary) and az-corals maintained their diversity in the Late Miocene and further increased it in the Pliocene and Pleistocene (*40*). A decoupling of the responses of shallow and deep-water corals may have occurred within the pre-evaporitic Messinian, and the revised coral dataset (*34*) confirms this hypothesis, highlighting a marked modification of the scleractinian coral pool of the Mediterranean during the Late Miocene and Early Pliocene due to the loss of the reef-building and shallow-water z-coral assemblages.

#### Ecophenotypic adaptations

Ecophenotypic adaptations were an additional impact of the pre-evaporitic Messinian environmental stress. Although the diversity of Sirenia did not change, they present a characteristic example of morphological changes due to the early Messinian restriction and change in the Mediterranean marine ecosystem (*57*). Sirenians ate seagrasses, and their tusk enamel δ^13^C before and after MSC reflected a shift in seagrass associations from *Posidonia oceanica* meadows in the Tortonian to more euryhaline forms with larger rhizomes like *Zostera* in the Messinian. Large-tusked sirenian species ate seagrass species with large rhizomes, which they extracted from the sediment, whereas species with small tusks ate leaves and shallow rhizomes (*58*). The dwarf size and large tusks of the endemic Mediterranean species *Metaxytherium serresii* are considered ecophenotypic adaptations due to the restriction of the habitat space and the change in the available food resources in the Messinian, which were reversed after the MSC, leading to the establishment of *Metaxytherium subappenicum* in the Pliocene (*57*).

#### Endemism

The high turnover and the low nestedness in all groups before and after the MSC (Fig. 3) reflect the importance of endemism in forming the Mediterranean biota. Oceanic endemic species were driven by the restriction of the connection with the Atlantic oceanic pool, whereas the Messinian neritic faunas were strongly affected by the establishment of the Paratethys-Mediterranean connection at 6.1 Ma that enabled the migration of species from the Paratethys to the Mediterranean and the evolution of new endemic species (*59*). Overall, the changing conditions throughout the Messinian triggered the appearance of new endemic species in the case for gastropods and bryozoans, whose species richness increases from the Tortonian to the Messinian (Fig. 2). Even so, species turnover is greatest for all groups between the Messinian and the Zanclean. Only for the planktonic groups, dissimilarity is below 50% between all comparisons (Fig. 3) because the Mediterranean and Atlantic surface waters were well-connected, and endemism is generally low in these groups. Nevertheless, both pre- to post-MSC comparisons demonstrate higher species turnover than the comparison between the Tortonian and Messinian plankton communities. For bivalves and gastropods, habitat fragmentation in the Zanclean due to the separation of the Adriatic from the Tyrrhenian Seas contributed to new endemics, thus increasing turnover (Fig. 3, H and I). In contrast, for ostracods, the recolonization from the Atlantic at the end of the MSC took place gradually, with only a few bathyal, opportunistic species recorded in the basal Zanclean (*60*). Possibly, new littoral endemic Mediterranean ostracod species also appeared later, in the Piacenzian, which would explain the low Zanclean species richness in comparison to the Tortonian and pre-evaporitic Messinian ones (Fig. 2E). Moreover, the Zanclean gastropod record is enriched with small-sized forms (e.g., fissurellids, trochids, rissoids, and cerithiids), whose absence in the Late Miocene may be attributed to preferential loss by dissolution, difference in study design (few sieved samples are available), and difficulty in extraction of the specimens due to increased cementation.

#### Regional versus global patterns

Some bioevents may be attributed to first-order regional-scale oceanographic episodes. In general, biotic immigration and isolation cycles have been shown to increase global biodiversity (*61*). This is not supported by a scenario where global extinctions of mollusc species were facilitated by the MSC that prevented these organisms from seeking refuge in the Mediterranean during Messinian glacials [e.g., (*33*)]. The most notable signal comes from the presence in the Early Pliocene of taxa indicative of deep psychrospheric water masses from the Northern Adriatic, reflecting an estuarine circulation regime at Gibraltar [e.g., (*41*)]. Specifically, the shark remains in the lower Zanclean sediments belong to species, which, on the whole, comprise a deep-water paleocommunity depicting a high degree of “oceanization” of Mediterranean Sea. At the same time, the high diversity and the turnover from archaic (i.e., Eurhinodelphinidae and Squalodontidae) to modern (i.e., Balaeonopteridae, Kogiidae, and Pontoporiidae) families of cetaceans coincides with the Middle–Late Miocene global diversity peak that may be related to high, diatom-driven marine productivity combined with climatic change (*62*). Printed over a gradual declining trend associated with climate cooling, the observed negative peak of the global marine mammal diversity in the Messinian could have been caused by the MSC (*63*), or it could be an artifact due to lack of data for this age for the Mediterranean Basin (*64*). Several cases support the dominance of global rather than regional patterns determining Mediterranean marine mammal diversity. For example, oceanic dolphins (Delphinidae) first appeared at the beginning of the Pliocene not only in the Mediterranean (*62*) but also in the North Atlantic. Nevertheless, within the Mediterranean, oceanic dolphins were more diverse, which could be due to the increased research effort in this region. Alternatively, this high diversity combined with an apparently high degree of species- and genus-level endemicity could be interpreted as an indirect consequence of the MSC: Delphinids may have recolonized the Mediterranean at the beginning of the Pliocene from the Atlantic Ocean, occupying the ecological niches available after the extinction/extirpation of the Miocene cetacean fauna (*62*).

### Limitations

The distribution of the occurrences (figs. S1 and S2) is highly uneven both geographically and in terms of sedimentary facies representation, leading to sampling bias (*65*, *66*). First, the fossil record analyzed here (*34*) was derived from paleontological studies conducted since the 19th century and the beginning of the 20th century, but these were constrained by the socioeconomic and political conditions, favoring sampling in the European Mediterranean countries. Second, biodiversity estimated from the paleontological record depends on the volume and area of the sedimentary rocks exposed and potentially accessible for studies of their fossil content (*67*). This is, in turn, related to the extent and method of sampling, which can also lead to large biases. In addition to the inherent sampling bias in paleobiodiversity studies (*68*), sampling approaches and methods changed markedly during the past 120 years. For large animals, for example, such as marine mammals and fishes, selective sampling in the early 20th century focused only on large specimens, easy to retrieve and handle, and often disregarding smaller fossils (*69*).

Environmental factors (such as currents and turbidity, pH, hydrostatic pressure, and sedimentation rate) largely determine the quality (and quantity) of the fossil record through taphonomic preservation (*70*). For instance, an important part of the recorded Zanclean diversity of bivalves and gastropods is contributed by small-sized aragonitic forms, which could be underrepresented in the Late Miocene collections because of preservation and/or sampling biases due to the difference in facies representation before and after the MSC in the Mediterranean. Marl and sand facies, which facilitate the preservation of small taxa, are common in the Zanclean [e.g., (*71*)] but nearly absent in the Late Miocene. Coral reef environments, which formed major parts of the Late Miocene Mediterranean coasts but became absent in the Zanclean, exhibited high cementation, and aragonitic shells were easily dissolved there [e.g., (*65*)].

Certain records are simply inaccessible to scientists for practical reasons: For example, the sediment cores obtained by deep-sea drilling expeditions in the Mediterranean yielded important records of microfossil occurrences for the Pliocene, but they neither could be used to obtain information about larger animals because of their dimensions nor could provide relevant information in pre-evaporitic records until recently. Consequently, for individual groups, the occurrences are often unevenly distributed between the three time intervals, leaving intervals with very few or even no occurrences.

For some groups, such as the ostracods, the available data cover all intervals, regions, and environmental settings adequately. However, there are several examples where a combination of the above biases has led to unevenly distributed fossil records. Even in the few areas with continuous composite stratigraphic sequences, such as Sicily and the Nile Delta, the diversity patterns through time within individual groups can be very different. For example, in the case of dinoflagellate cysts, sampled with the same methodology throughout the Tortonian-Zanclean stratigraphic interval, species richness is highest during the Messinian in the record of the Nile Delta but lowest in Sicily record, compared to the Tortonian and Zanclean.

The rarefaction method used in the calculation of diversity indices to (at least partly) overcome these sampling biases does not allow us to quantitatively evaluate several records due to this lack of sufficient number of occurrences (arbitrarily set at 15 occurrences in the present analysis) in one or more of the time intervals for a given taxonomic group. Groups with few occurrences within an interval (Figs. 1 and 2) (*34*) should be targeted for further systematic paleontological studies.

Last, we excluded from the present study the limited fossil record from the MSC stages because it was not sufficient for this type of analysis, and, therefore, we cannot quantify in absolute terms the Mediterranean marine biodiversity during the MSC. Overcoming this limitation through further research in the future would be challenging, as taphonomic reasons certainly contributed to the scarcity of the fossil record, and evaporite deposition is not conducive to carbonate or siliceous fossil preservation (*37*). In addition, the abundance of marine organisms was probably low as well. Halophiles have been identified from the evaporites (*72*) and the intercalated marls of the first stage of the MSC (5.86 to 5.6 Ma), potentially inhabiting the bottom part of the water column (*73*). Simultaneously, marine fossils are rare to uncommon, and their presence has been debated, particularly in the final MSC stage, because they co-occur with fossils of brackish-water species (*23*, *37*).

### Ecologic, oceanographic, and climatic implications of biodiversity change

The biodiversity changes, which are quantified here at the taxonomic level and on the basis of the current status of the fossil record (Table 1), point toward an ecological crisis in the Mediterranean during the Messinian, even before and peaking at the MSC. Although controversy is still high on the true nature and evolution of the MSC events [e.g., (*74*, *75*)], direct and indirect ecosystem consequences of the MSC cannot be denied. The interplay between climate and connectivity changes at the timescale of hundreds of thousands of years resulted in a combination of responses at all spatial scales and levels of organization, from lifestyle changes (e.g., in the case of whales who could no longer migrate into the Mediterranean during the MSC) to evolutionary responses (e.g., the endemic Mediterranean species emerging during the pre-evaporitic Messinian in many groups). The outcome is a disruption and reorganization of the ecosystem already in the pre-evaporitic Messinian and even more in the Zanclean (Fig. 1).

To advance beyond these outcomes, future research should explore the implications of these changes for food webs, ecosystem structure, and function and for biogeochemical cycles. Functional diversity refers to the traits and niches filled by species, which essentially control how diversity influences the functioning of the ecosystem (*76*). For example, communities with the same species richness (a measure of taxonomic diversity) may include species with vastly different traits (e.g., pelagic versus demersal lifestyle), who occupy a different ecological niche in the ecosystem, thus forming very different ecosystems. It is possible that the MSC resulted in or facilitated changes in functional, in addition to taxonomic, diversity in the Mediterranean marine ecosystem. The most characteristic is the case of corals, where tropical, reef-building z-corals disappeared completely from the Mediterranean after the MSC (*40*). Furthermore, the functional composition of the marine biota determines the structure of the food web and, thus, the flow of energy and nutrients. Critical intervals, such as the Messinian for the Mediterranean Sea, involve perturbations of the biogeochemical cycles. Another possibility is that the Messinian decrease in mesopelagic fish diversity may have led to a decline in carbon export efficiency. These and other hypotheses should be tested, which would have important implications for the subsequent evolution of the Mediterranean environment.

## MATERIALS AND METHODS

### Experimental design

The dataset that was used contains 22,989 fossil occurrences of 4933 species, including some occurrences that have been considered reworked (*34*). We excluded these reworked occurrences from the present analysis. Each occurrence was designated by the unique combination of a taxon found at a specific locality in one of the three intervals: Tortonian, pre-evaporitic Messinian, or Zanclean. This assignment of each occurrence to a time interval was based on the most up-to-date literature on the chronostratigraphy of the sediments holding the corresponding fossil remains, as indicated in the revised dataset (*34*, *35*).

To examine the biodiversity changes observed for each group of organisms through time within Mediterranean regions, we distinguished the fossil localities into three marine regions on the basis of their paleogeographic placement in the Western Mediterranean, the Eastern Mediterranean, and the Po Plain–Northern Adriatic, following the now accepted paleogeographic data (*77*, *78*). The Po Plain–Northern Adriatic region developed as a paleoceanographic subbasin of the Mediterranean in the Tortonian–early Messinian, as evidenced by its distinct strontium isotopic signature (*79*) and the absence of halite deposits. We placed the Tortonian records from Piedmont and the Po Plain within the Po Plain–Northern Adriatic region as well, attempting to test the hypothesis that this region held an already distinct marine biota in the Tortonian. On the basis of the Late Miocene paleogeographic evolution of Calabria and Sicily (*80*–*82*), we included the records from these areas as part of the Eastern Mediterranean because the marine connection with the Western Mediterranean was located near its present location, possibly along present southern Sicily (*83*) or at the Sicily Channel [Malta Plateau; (*84*)].

We conducted the present analyses for all corals and, separately, for z-corals and az-corals. However, because there are no z-corals in the Mediterranean in the Zanclean and the number of genera and species in the Tortonian and pre-evaporitic Messinian is too small for the analysis after subsampling, we illustrate here the results for all corals together (Figs. 1 to 3) and for az-corals only (fig. S9). A list of the genera present in the Mediterranean during the Tortonian-Zanclean belonging to az- or z-corals is included in (*34*). In the cases where a genus includes species of both categories, the attribution for the present analysis was decided on the basis of the environmental setting where the coral remains were found, as reconstructed from the accompanying fauna and the lithology.

### Statistical analysis

Biodiversity can be evaluated across scales by evaluating alpha, beta, and gamma diversity (*85*). Alpha diversity refers to species richness, which is the number of species within a community. Beta diversity represents the amount of differentiation between communities, which may be due to (i) species turnover, which is the replacement of species by novel (and different) ones, independent of species richness; and (ii) nestedness, resulting from species loss through extinction (*86*). Nestedness, as an independent property of communities within an ecosystem, shows the difference between the faunas compared in terms of loss of species: When two faunas exhibit low nestedness (i.e., they are not nested), one is not a subset of the other (*87*). Gamma diversity refers to the total biodiversity across a larger geographic area [e.g., (*88*)].

We calculated (i) species and genus richness in the Tortonian, the pre-evaporitic Messinian, and the Zanclean of the Mediterranean as a whole and of its three regions separately; and (ii) beta diversity comparing the different time intervals for the entire Mediterranean and the three regions at both species and genus level (*89*, *90*). We calculated the metrics: richness (i.e., number of species or genera), Søerensen index (total dissimilarity between the compared intervals), Simpson index (dissimilarity due to turnover), and nestedness (*39*, *87*). To overcome potential bias in our biodiversity estimates due to the differences in sampling effort between the regions and intervals, we used an 80% rarefaction, which has been shown to produce robust results (*91*). In practice, we subsampled the record of the intervals that are compared to the 80% of the lowest number of occurrences between them, and we used the subsamples for calculating the diversity indices. Subsampling was repeated 10,000 times. The data for sharks and marine mammals were not analyzed statistically separately given the limited number of recorded specimens and taxa and because cetaceans were mostly represented by poorly preserved diagnostic material that did not allow determination below the family level.

## Supplementary Materials

### This PDF file includes:

Supplementary Text Figs. S1 to S11 Table S1 References
