# Supplementary material for: Late Miocene transformation of Mediterranean Sea biodiversity
Source: Sci Adv. 2024 Sep 25;10(39):eadp1134. doi: 10.1126/sciadv.adp1134 (PMC11423897; doi:10.1126/sciadv.adp1134)
Supplement: Supplementary file 1 — Supplementary Text Figs. S1 to S11 Table S1 References [file sciadv.adp1134_sm.pdf]

Science Advances
AAAS

Supplementary Materials for

Late Miocene transformation of Mediterranean Sea biodiversity

Konstantina Agiadi *et al.*

Corresponding author: Konstantina Agiadi, konstantina.agiadi@univie.ac.at

Sci. Adv. **10**, eadp1134 (2024)
DOI: 10.1126/sciadv.adp1134

This PDF file includes:

Supplementary Text
Figs. S1 to S11
Table S1
References

Additional background and results by taxonomic group

Dinocysts

The protoperidinioid/ gonyaulacoid cysts ratio in the record (Table S1) suggests higher productivity and hydrological surface circulation in the Western Mediterranean during the Tortonian, than in the pre-evaporitic Messinian, but lower during the Zanclean, probably reflecting the restriction of the Tortonian wide connections with the Atlantic. The ratio of protoperidinioid over gonyaulacoid cysts is an indicator of the abundance of cysts produced by heterotrophic versus autotrophic dinoflagellates (*97*), and it is considered a measure of primary productivity. However, information on preservation can also be contained in this index (*98*), as well as bias due to the sample processing method (*99*). Moreover, the cysts of protoperidinoid dinoflagellates are known to be sensitive to oxidation (*100*).

Planktic foraminifera

Shifts in planktic foraminifera abundances have often been associated with lithological alternations, which in turn have been correlated with precession (*101*). Cold/eutrophic planktic species dominate the Late Miocene homogeneous marls deposited during insolation minima, whereas the assemblages are dominated by warm/oligotrophic species at times of insolation maxima accompanied by the deposition of laminated marls (*20*, *102*, *103*). Selli (*104*) interpreted some "dystopic faunal elements" as signs of the beginning of the salinity crisis. The most important bioevent at the Tortonian/Messinian boundary (7.25 Ma) is the replacement of right-coiled *Globorotalia menardii* form 5 by left-coiled representatives of the *Globorotalia miotumida* group (*Globorotalia miotumida* and *Globorotalia conomiozea* along with their intermediates *Globorotalia miocenica mediterranea, Globorotalia saphoae,* and *Globorotalia dalii*) (*105*). The Messinian stratigraphic distributions of the species *Globoturborotalita nepenthes, Globigerinoides trilobus* and neogloboquadriniids show distinct intermittent presence patterns across the basin (*20*, *103*). Unkeeled deep-dwelling globorotaliids were sparsely present after 6.72 Ma, with their disappearance being attributed to the increase of salinity at or beyond their tolerance limits (*20*). According to Kontakiotis et al.(*15*), high-salinity conditions (SSS > 40‰) were developed since 6.9 Ma in the Eastern Mediterranean, resulting in thermal and salinity stressful conditions, which were reflected in a less diversified planktic fauna (often containing only 3–4 species) and increased abundances of taxa considered tolerant to increased salinity. This decrease in planktic foraminifera diversity started at 6.9 Ma and peaked after 6.7 Ma (*103*).

The intermittent occurrence of almost monospecific *Turborotalita quinqueloba/ Turborotalita multiloba* assemblages (*102*, *106*), and/or *Orbulina universa* blooms (*103*, *107*), and the overturn in the most abundant shallow-dwelling species (decrease of *Globigerinoides obliquus* group and increase of *Globigerina bulloides* group have been interpreted as indicative of the progressive restriction of the Mediterranean that produced a more eutrophic environment related to the salinity increase. It is worth noting that all these species can tolerate hypersaline surface waters (*108*), and particularly the small-sized opportunistic species *T. quinqueloba* and *T. multiloba* can dominate over others in such highly stressed environments (*103*). The similarly discontinuous distribution pattern of neogloboquadriniids was probably controlled by changes in the deep chlorophyll maximum, which was absent in dry periods, but expanded (though not productive) during wet periods (*20*).

Benthic foraminifera

Despite the overall increasing species richness from the Tortonian to the pre-evaporitic Messinian in the entire Mediterranean basin, benthic foraminifera assemblages became oligospecific in the Western Mediterranean after 7.17 Ma, presumably due to changes in bottom-water oxygenation: high-oxygen benthic foraminifera species (e.g., *Siphonina reticulata,*

Cibicidoides italicus, *C. kullenbergi*) were replaced by the species *Oridorsalis umbonatus*, *Sphaeroidina bulloides*, *Uvigerina peregrina*, *Gyroidinoides* spp., *Melonis* spp. (*17*, *18*, *109*), which are associated with reduced oxygen conditions and/or increased organic matter content (*110*). The benthic foraminifera records from the Po Plain–Northern Adriatic sections Monte del Casino and Trave show analogous changes in the assemblages after 7.17 Ma (*111*). In the Eastern Mediterranean, such changes were more intense and, in addition to the disappearance of the high-oxygen taxa, the new assemblages were dominated by buliminids (e.g., *B. elongata*, *B. subulata*, *B. aculeata*), bolivinids (*B. scalprata miocenica*, *B. dilatata*, *B. plicatella*, *B. spathulata*), and uvigerinids (e.g., *U. peregrina*, *U. cylindrica*, *U. striatissima*), reflecting high organic-carbon flux to the sea floor, increased salinity and/or anoxic/dysoxic conditions (*55*, *112*, *113*). Benthic foraminifera communities were additionally impacted by the further deterioration of bottom-water conditions at 6.7–6.8 Ma, when deep-water ventilation was further reduced and organic carbon flux to the sea floor increased in both the Western (*32*, *103*, *114*) and the Eastern Mediterranean (*115*). Additionally, in some localities, the concomitant appearance of shallow-water species such as *Elphidium* spp., *Cibicides lobatolus*, *Discorbis* spp., *Asterigerina planorbis*, *Rosalina* spp., and *Valvulineria bradyana* is observed (*55*, *110*, *112*).

With the Zanclean reflooding, normal and well-oxygenated conditions were gradually re-established in the Mediterranean basin (*116*) allowing the return of the high-oxygen species that disappeared since 7.17 Ma (e.g., *Siphonina reticulata*, *Cibicidoides italicus*, *C. kullenbergi*). In particular, the recolonization by *S. reticulata* in the Mediterranean appears to be a nearly synchronous event (*117*, *118*), recorded at the beginning of the MPI 2 biozone (*30*, *118*). The species turnover at the base of the Pliocene was stronger than the one recorded after the Tortonian/Messinian boundary (Fig. 3D).

<u>Ostracods</u>

Ostracods have been very important in reconstructing the paleoenvironmental conditions in the Mediterranean particularly in the pre-evaporitic Messinian and the Lago Mare stages, particularly when salinity variations are investigated. The only known species present in the Neogene and Quaternary of the Mediterranean that are considered capable of tolerating great variations in seawater salinity, from hypo- to hypersaline conditions, belong to the genus *Cyprideis*. The ecology of the extinct *Cyprideis* species has been inferred based on the present knowledge of the ecology of the extant species *Cyprideis torosa*, which still inhabits the Mediterranean. *Cyprideis torosa* is an euryhaline generalist species, which is typical of brackish environments (*119* and references therein), but it has been reported both from hypohaline athalassic conditions (*120*), and from salt pans (*121*, *122*). Its ability to survive and even thrive in such extreme conditions has been attributed to extreme osmotic regulation (e.g., *123*, *124*), and it represents an excellent paleosalinometer since the morphology of its carapace differs depending on the salinity conditions (*121*, *125*–*127*). *Cyprideis torosa* is dominant in hyposaline environments, where it is accompanied by both halophilic species from continental input (e.g., *Heterocypris salina*, *Darwinula stevensoni*, *Candona angulata*, *Ilyocypris* spp.) and marine ostracods tolerating lower salinities (e.g., *Loxoconcha elliptica*, *Leptocythere* spp., *Basslerites berchoni*, *Cytherois fisheri*). When conditions become hypersaline, *C. torosa* is the only ostracod that is present, in monospecific assemblages. Up to around 50 ‰, it is commonly accompanied by the benthic foraminifer *Ammonia tepida*. None of the *Cyprideis* species found in the Tortonian and pre-evaporitic Messinian of the Mediterranean have been associated with strictly hypersaline, but rather with brackish environments (*121*). Only during the last stage of the MSC, *Cyprideis agrigentina* assemblages accompanied by *Ammonia tepida* make up the early Lago Mare biofacies (*128*) or "*Cyprideis* zone" (*129*) in both marginal and deep-sea sites across the Mediterranean (*130*, *131*, *121*).

Corals

Before the MSC, the most abundant genera were *Tarbellastrea*, *Solenestrea* and *Siderastrea*, while *Porites* was the main frame-builder species of pre-evaporitic Messinian reefs (*93*, *94*). Except *Solenastrea*, these genera also had the widest geographic and stratigraphic distribution suggesting that they were able to adapt to diverse paleoenvironmental conditions.

Bivalves

Looking only at Ostreida and Pectinida (large-sized, calcitic shells, with generally better preservation and greater stratigraphic and geographic distributions), their species richness drops from the Tortonian to the pre-evaporitic Messinian.

Gastropods

There is a turnover in families of medium-sized gastropods (Neogastropoda) from communities rich in species of the family Clavatulidae. In contrast, other apparent faunal changes among carnivore gastropods such as the sharp increase in number of species of the families Mangeliidae and Raphitomidae (Neogastropoda), Eulimidae (Caenogastrpopoda) and the parasitic family Pyramidellidae (Heterobranchia) in the Zanclean may be attributed to the biases of the record (see *Limitations* section in the main text) — few occurrences of these taxa in the Late Miocene, probably due to preservation and sampling bias. Moreover, the biodiversity results are driven by changes in the Po Plain–Northern Adriatic region, because the fossil record (*34*) does not contain sufficient occurrences from all stages in all three regions to conduct the analysis.

Bryozoans

The Mediterranean fossil record for the Zanclean is limited to a few localities (*34*). The most abundant and diverse order of bryozoans, the Cheilostomes, is the best studied and represented in the Neogene Mediterranean deposits, corresponding to about 80% of the analyzed faunas. A relatively high proportion of cheilostome species (68%) are common between the Messinian and the Pliocene (*132*). Compared to the present-day, about 26% of present-day Mediterranean species appeared before or during the Messinian, whereas 17% are endemics (*132*). Apart from this marked continuity of the taxa, a progressive disappearance of thermophilic Tethyan relics has been observed (e.g., *Biflustra savarti*, *Emballotheca*, *Metrarabdotos*, *Nellia*, *Steginoporella*) and attributed to the Late Miocene global cooling, whereas some modern taxa of Atlantic origin only appeared in the pre-evaporitic Messinian (e.g., *Cryptosula pallasiana, Schizotheca fissa*, *Scrupocellaria scrupea*), mostly in the Western (*133*) and less so in the Eastern Mediterranean (*134*).

Echinoids

In the Tortonian, the Mediterranean echinoid assemblage was rich and dominated by shallow-water tropical-subtropical species, such as the genera *Clypeaster* and *Echinolampas* (*135*). The stressful environmental conditions in the pre-evaporitic Messinian would be expected to have an impact on echinoids, which are exclusively marine invertebrates, intolerant of great salinity fluctuations that are never found in freshwater (*136*) and rarely can tolerate even moderate salinity changes (*137*).

Bony fishes

Otolith and skeletal findings were combined to obtain the Tortonian–Zanclean fossil record of bony fishes (*34*), which collectively represent both the shallow and deep environments before and after the MSC within the Mediterranean and the three regions. The Tortonian and pre-evaporitic Messinian bony fish record of the Mediterranean (*34*) comprised species of Mediterranean–Atlantic distribution (*138*) and several with Paratethyan-affinity (*139*, *140*). In the

pre-evaporitic Messinian, the fossil record shows the extirpation of several pelagic species that were common in the Mediterranean in the Tortonian, including mesopelagic fishes of the family Myctophidae such as *Benthosema fitchi, B. glaciale, Lobianchia gemellarii, Myctophum punctatum, Notoscopelus bolini* and *N. resplendens* and deep-water fishes such as *Coelorinchus* spp., *Trachyrincus* spp. and *Verilus* spp. (*139*, *141*, *142*). Notably, after 6.8 Ma, otolith isotopic data have indicated that benthic fish growth was severely hampered by a combination of high salinity stratified bottom waters with high temperature fluctuations, leading to their final disappearance from the sea bottoms at least in the Eastern Mediterranean, even before the MSC onset (*114*). All of these species re-established in the Mediterranean after the MSC. Additionally, several endemic Mediterranean species appeared during the pre-evaporitic Messinian, e.g. myctophids such as *Ceratoscopelus dorsalis* and *C. miocenicus* (*143*, *144*) and benthic gobies such as *Buenia affinis* and *Caspiosoma lini* (*140*, *143*); most, but not all, of these endemics disappeared in the Zanclean. Apart from the reintroduction to the Mediterranean of common Tortonian species in the Zanclean, we also observe the first appearance in the basin of common extant benthic species *Chromogobius zebratus, Gobius guerini, Gobius geniporus, Gobius niger, Lesueurigobius sanzi, Zebrus zebrus* and *Zosterisessor ophiocephalus* (*140*, *145*, *146*).

<u>Sharks</u>

The scarcity of Messinian records of elasmobranchs complicates the reconstruction of the Late Miocene Mediterranean fauna (*34*). That said, the Messinian assemblage from Algeria (*147*) strongly resembles other central Mediterranean Tortonian assemblages (e.g., *148, 149*) in terms of taxonomic composition. Most of the Zanclean assemblage is represented by relic Miocene taxa. In the lowermost Pliocene, published occurrences of taxa such as *Carcharocles megalodon* and *Hemipristis serra* hint at the persistence of the Miocene structure of the Mediterranean fauna during the early Zanclean (*150*). Other relic Miocene species (e.g., *Megascyliorhinus miocaenicus*, *Pachyscyllium dachiardii, Pachyscyllium distans*, *Cosmopolitodus plicatilis* and *Parotodus benedeni*) even persisted in the Mediterranean until the mid-Pliocene at least (*151, 152*). In the Late Miocene and Early Pliocene, *Carcharhinus* was likely the most speciose shark genus in the Mediterranean. The diverse appearance of the Pliocene *Carcharhinus* assemblage is seemingly due to the in-depth revision of the Pliocene carcharhines provided by Marsili (*153*). On the other hand, most Miocene carcharhines were attributed to the 'wastebasket' fossil species *Carcharhinus priscus* and *Carcharhinus egertoni* (*154*). While the former has been recently redefined on the basis of Agassiz' types by Reinecke et al. (*155*), a comprehensive revision of the Miocene Mediterranean teeth of *Carcharhinus* is still needed.

No strong breaks other than those observed globally can be recognized for the Mediterranean elasmobranch faunas around the Miocene/Pliocene transition. The lowermost Pliocene nearshore assemblages (*150, 156*) are, at various degrees, reminiscent of the Miocene faunas.

<u>Marine mammals</u>

The Late Miocene–Early Pliocene fossil record of marine mammals of the Mediterranean includes representatives of cetaceans (whales and dolphins), pinnipeds (seals) and sirenians (dugongs). The cetacean Tortonian Mediterranean record is quite diverse both among the echolocating toothed whales (Odontoceti) and the baleen whales (Mysticeti). Odontocetes are represented by Delphinida (dolphins), Physeteroidea (sperm whales) and Ziphiidae (beaked whales); while at least four mysticete families are present: Balaenidae (right whales), Balaenopteridae (rorquals), Eschrichtiidae (gray whales) and Cethotheriidae. The fossil record of cetaceans from the pre-evaporitic Messinian of the Mediterranean is limited to three specimens belonging to odontocetes, including a Phocoenidae (porpoise) and a Physeteroidea. The Zanclean and Tortonian cetacean fauna exhibit the same high-rank taxonomic composition. Nevertheless, the Early Pliocene features a greater diversity at the genus and species levels, which however may

reflect better preservation of the Zanclean specimens compared to the Tortonian ones. Interestingly, although several Miocene findings of Delphinidae (oceanic dolphins) have been reported from the Mediterranean, and the unambiguous fossil record of this odontocete family is not older than the Zanclean.

All determinable fossil remains of sirenians in the Late Miocene–Early Pliocene Mediterranean belong to dugongid genus *Metaxytherium*, which is represented by three putative chronospecies: the medium-sized *M. medium* (Tortonian), the small-sized *M. serresii* (latest Tortonian–earliest Zanclean), and the large-sized *M. subapenninum* (Zanclean). Although the fossil record of pinnipeds from Miocene Mediterranean deposits is extremely fragmentary (*157*), it suggests the continued presence of the seals Monachinae from the Early Miocene to the present day. Based on the Messinian record, which only consists of *Messiphoca mauritanica* from Algeria (*158*), it can be speculated that the Mediterranean pinnipeds were confined to even more restricted areas than they are today before the MSC onset. Most of the occurrences are indeed close to Gibraltar, which is reminiscent of the present-day Mediterranean distribution of *Monachus monachus* (*159*).

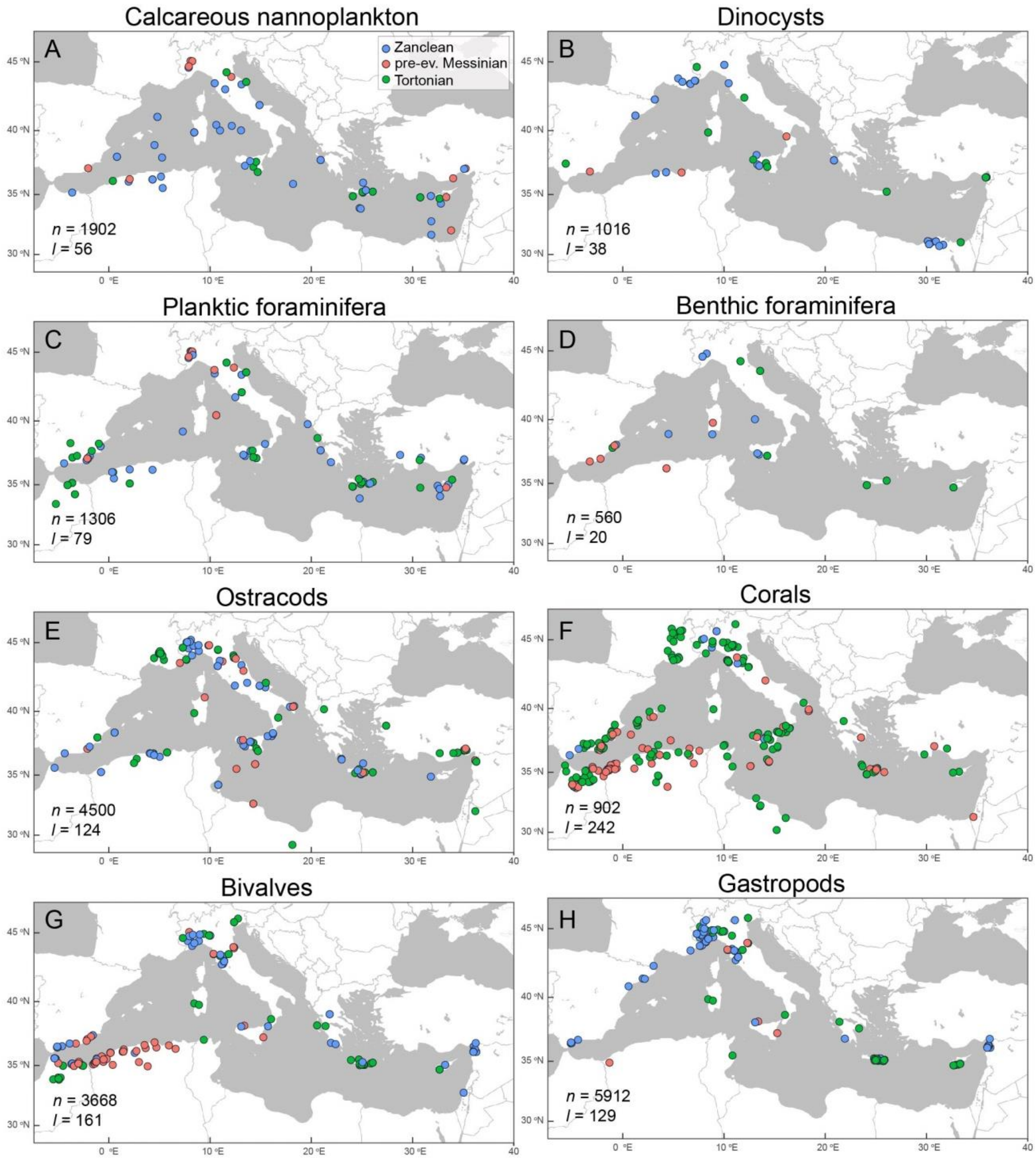

Fig. S1. Mediterranean map with the localities included in this record (*34*, *89*). A. Calcareous nannoplankton, B. Dinocysts, C. Planktic foraminifera, D. Benthic foraminifera, E. Ostracods, F. Corals, G. Bivalves, H. Gastropods. The maps were produced using ggmap (*160*). *n* indicates the number of occurrences in the dataset, and *l* indicated the number of localities.

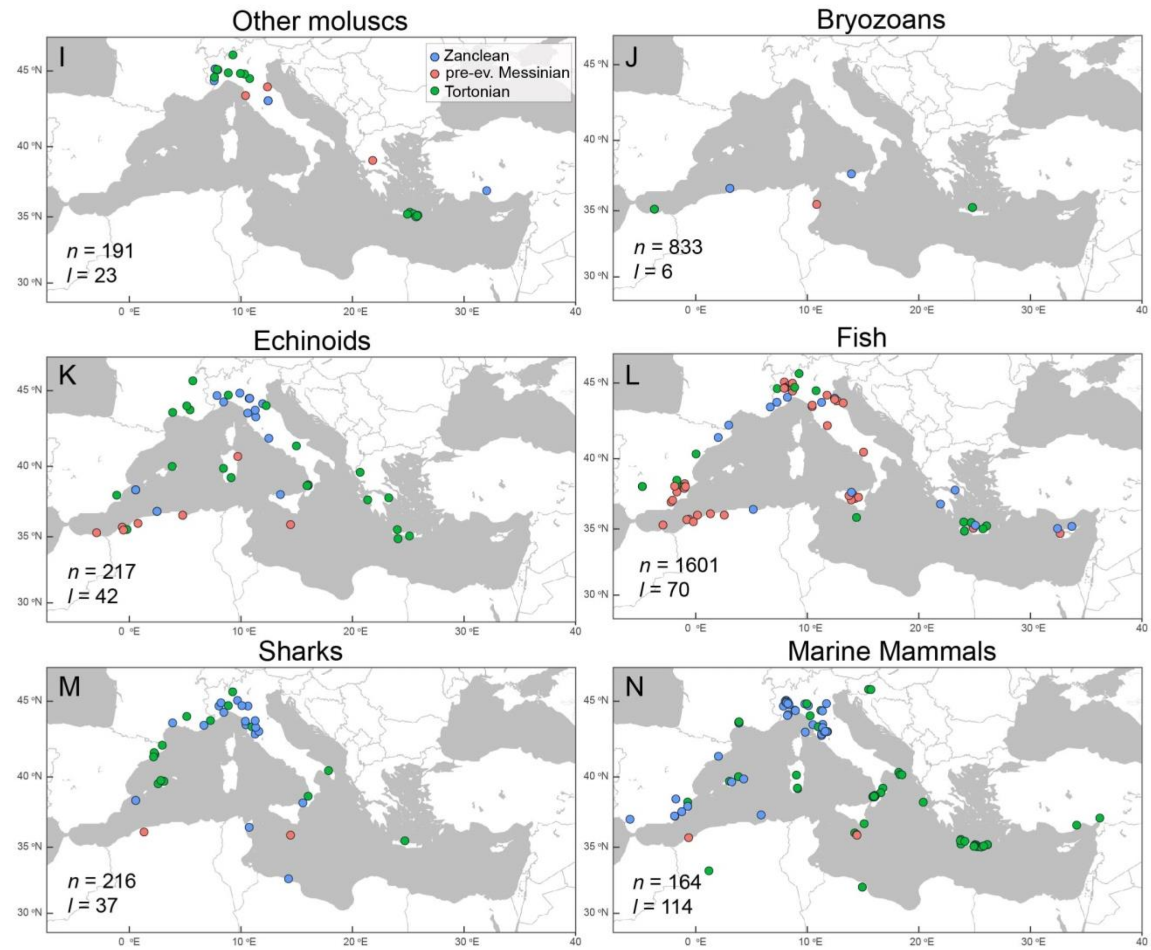

Fig. S2. Mediterranean map with the localities included in this record (*34*, *89*). I. Other molluscs (scaphopods, chitons, cephalopods), J. Bryozoans, K. Echinoids, L. Fish, M. Sharks, N. Marine mammals.

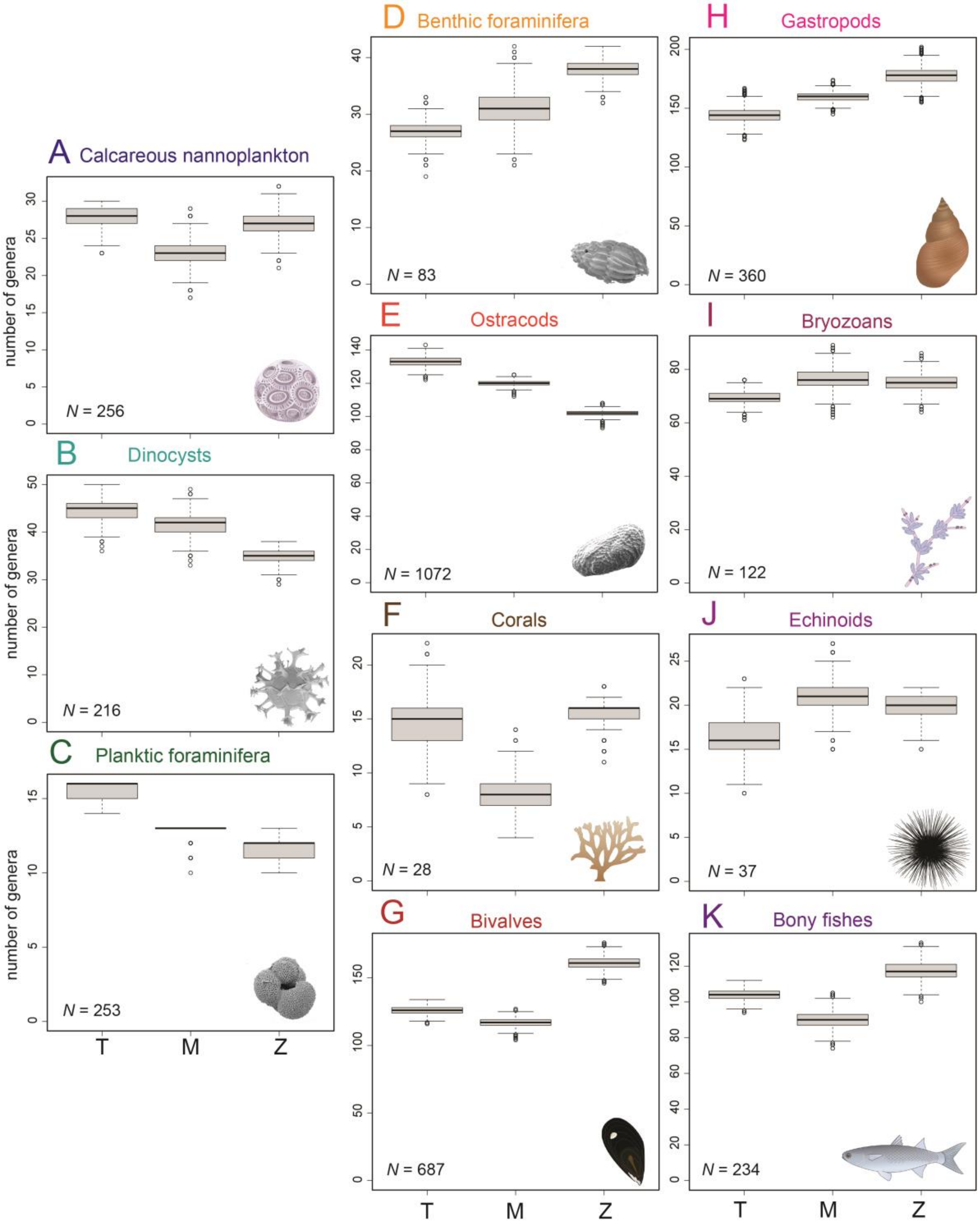

Fig. S3.

Changes in richness at the genus level of the Mediterranean Sea biota from the Late Miocene to the Early Pliocene by group of organisms. X-axes shows intervals: Tortonian (T), pre-evaporitic Messinian (M), and Zanclean (Z). The richness of corals in the Zanclean is only due to azooxanthellate corals, since zooxanthellate (reef-building) corals are not present in the Mediterranean after the MSC. Symbols for organisms obtained from the Integration and Application Network (ian.umces.edu/media-library). *N* indicates the number of occurrences after subsampling to 80% of the smallest sample, ten thousand times. The bold line indicates the median value, the box corresponds to the quartiles (values included fall within 25th and 75th percentiles of the data), whiskers are quartiles plus/minus 1.5 times the interquartile range.

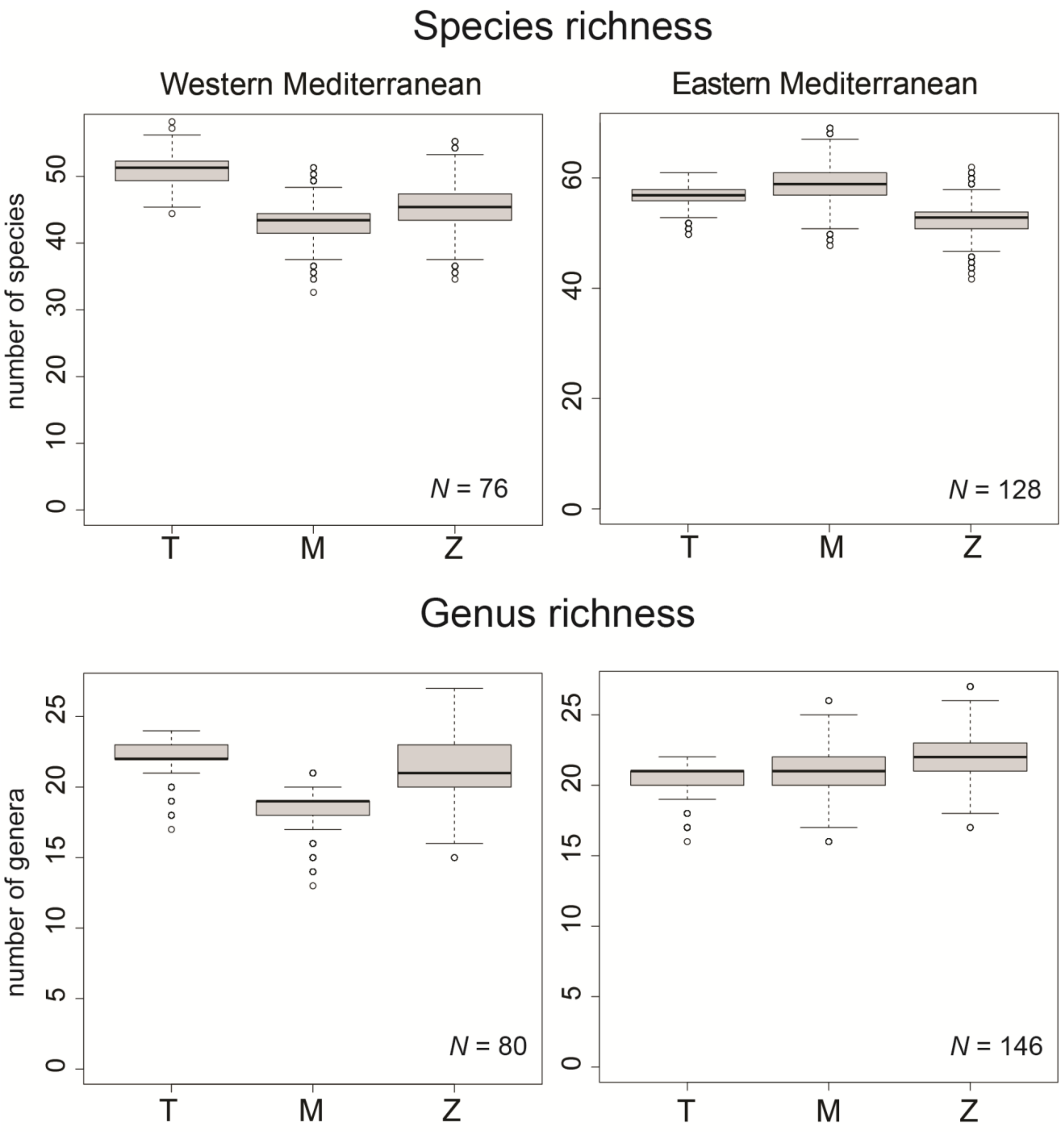

Fig. S4.

Calcareous nannoplankton species and genus richness through time in the Western and Eastern Mediterranean across the Tortonian (T), pre-evaporitic Messinian (M) and Zanclean (Z).

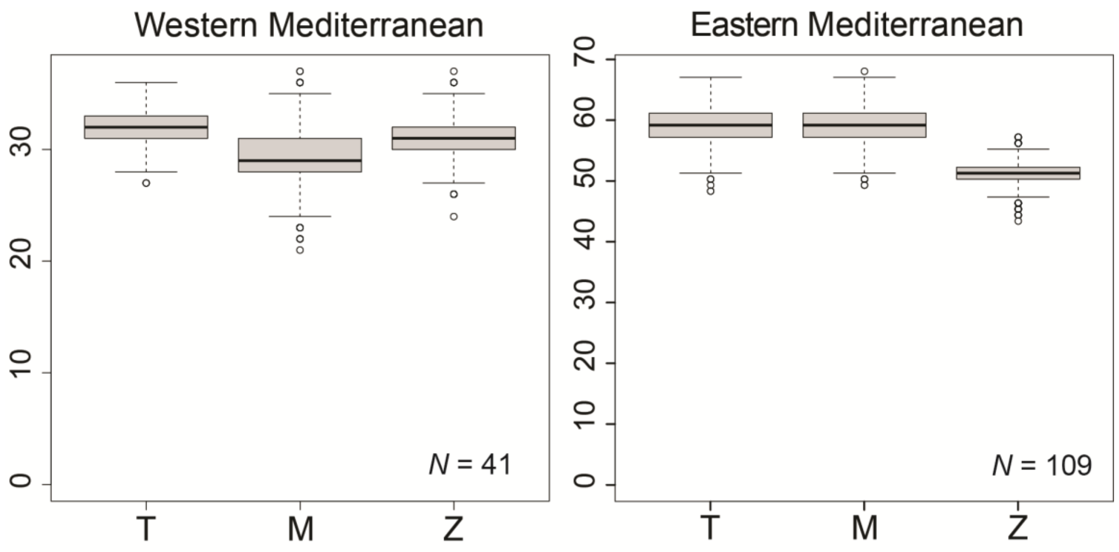

Fig. S5.

Dinocyst species richness in the Western and Eastern Mediterranean across the Tortonian (T), pre-evaporitic Messinian (M) and Zanclean (Z).

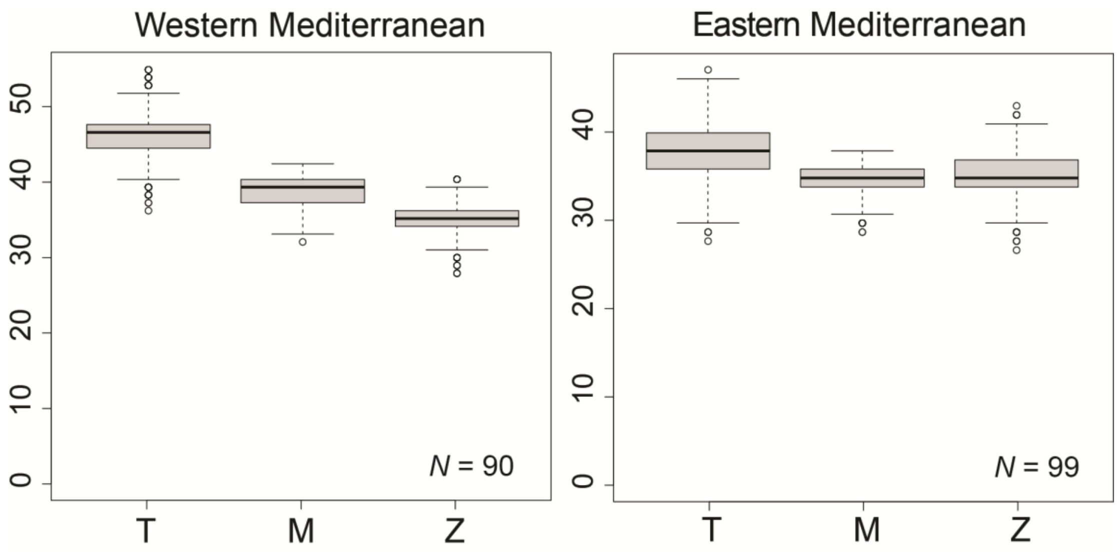

Fig. S6.

Temporal and spatial changes in species richness of the planktic foraminifera in the Mediterranean regions in the Tortonian (T), the pre-evaporitic Messinian (M), and the Zanclean (Z).

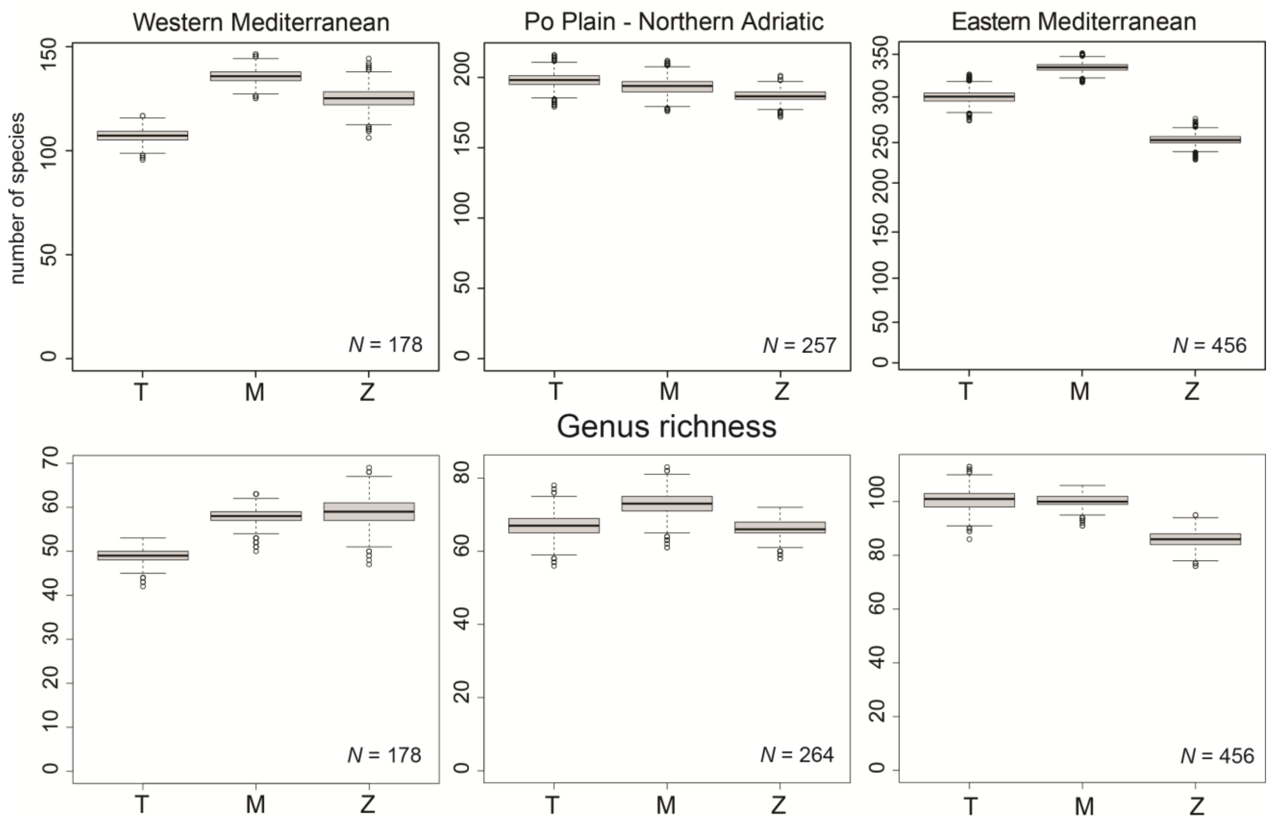

Fig. S7.

Ostracod species and genus richness in the Mediterranean regions in the Tortonian (T), the pre-evaporitic Messinian (M), and the Zanclean (Z).

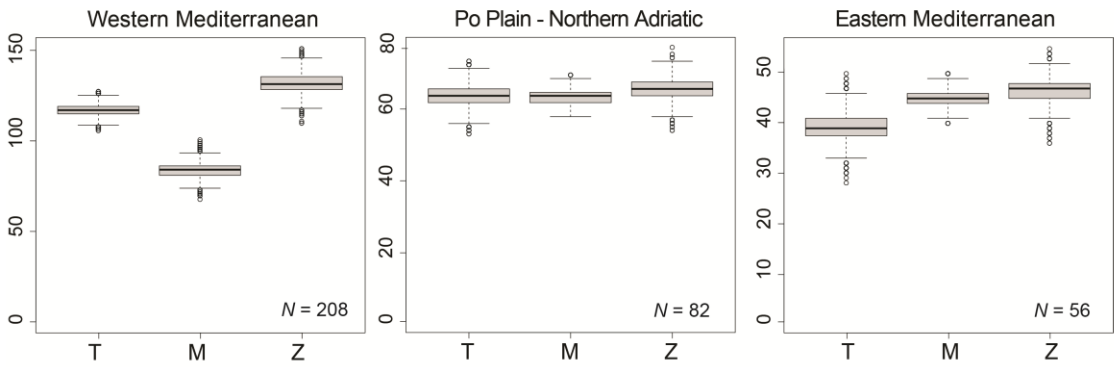

Fig. S8.

Changes in bivalve species richness of the Mediterranean Sea from the Tortonian (T), the pre-evaporitic Messinian (M) to the Zanclean (Z).

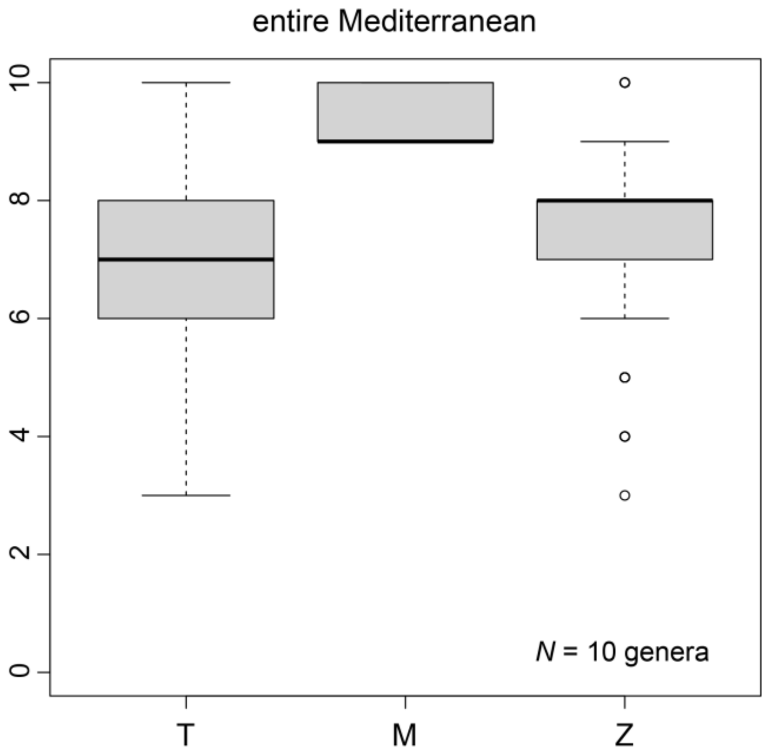

Fig. S9.
Changes in genus richness of azooxanthellate corals in the entire Mediterranean Sea from the Tortonian (T), the pre-evaporitic Messinian (M) to the Zanclean (Z).

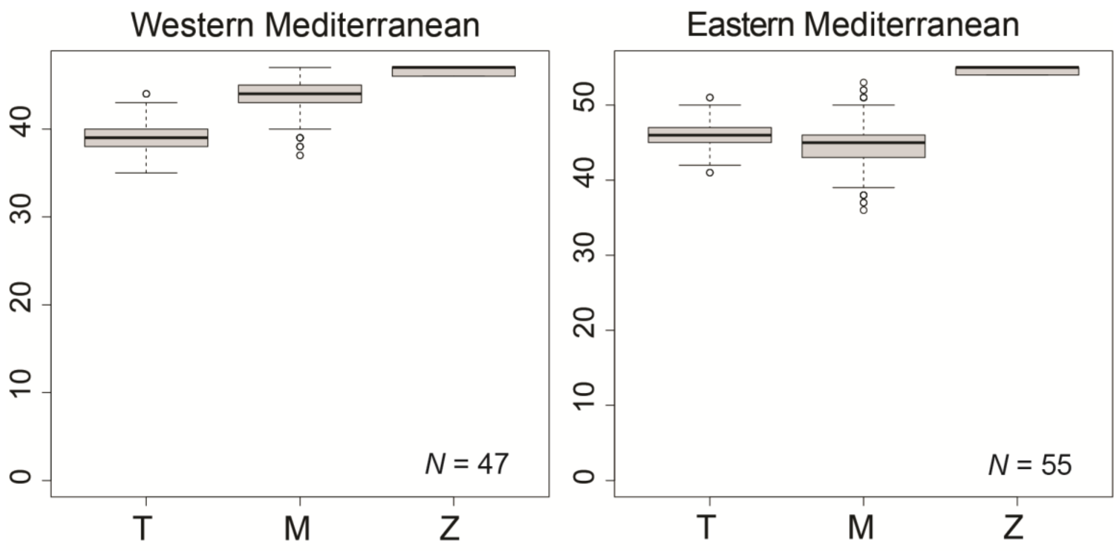

Fig. S10.

Species richness of the bryozoan fauna in the Western and Eastern Mediterranean in the Tortonian (T), the pre-evaporitic Messinian (M) and the Zanclean (Z).

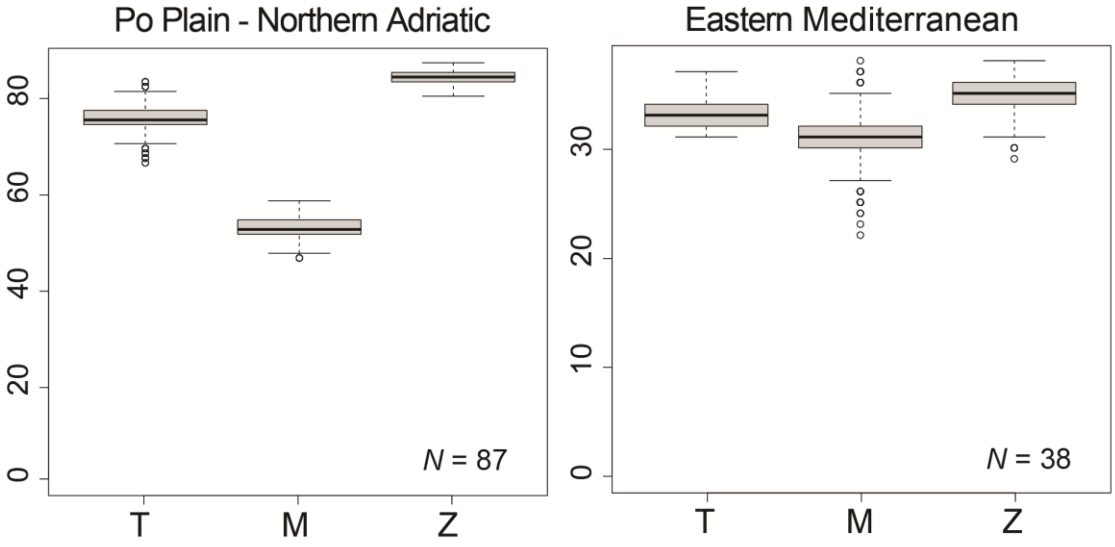

Fig. S11.

Species richness of bony fishes within the Eastern and the Po Plain-Northern Adriatic region in the Tortonian (T), the pre-evaporitic Messinian (M), and the Zanclean (Z).

	Western Mediterranean			Po Plain-Adriatic			Eastern Mediterranean		
	**T**	**M**	**Z**	**T**	**M**	**Z**	**T**	**M**	**Z**
Sequences	3	5	3	2	2	1	10	13	13
Species	53	77	52	41	42	21	90	92	73
Genera	24	25	20	24	23	11	38	37	26
Gonyaulacoid species	39	50	27	29	29	14	57	56	53
Protoperidinioid species	4	1	6	10	8	1	10	17	8
Goniodomacean species	8	8	2	3	3	2	4	4	3
Protoperidin/Gonyaulacoid ratio	10%	2%	18%	26%	22%	7%	15%	23%	13%

Table S1.

Summary of results obtained from bibliographic data for organic-walled dinoflagellate cysts for the Mediterranean and the three time intervals considered. T: Tortonian, M: pre-evaporitic Messinian, Z: Zanclean.

REFERENCES AND NOTES

1. D. P. Tittensor, C. Mora, W. Jetz, H. K. Lotze, D. Ricard, E. V. Berghe, B. Worm, Global patterns and predictors of marine biodiversity across taxa. *Nature* **466**, 1098–1101 (2010).

2. P. Lionello, L. Scarascia, The relation between climate change in the Mediterranean region and global warming. *Reg. Environ. Chang.* **18**, 1481–1493 (2018).

3. F. D. Por, One hundred years of Suez Canal—A century of lessepsian migration: Retrospect and viewpoints. *Syst. Biol.* **20**, 138–159 (1971).

4. P. G. Albano, L. Schultz, J. Wessely, M. Taviani, S. Dullinger, S. Danise, The dawn of the tropical Atlantic invasion into the Mediterranean Sea. *Proc. Nat. Acad. Sci. U.S.A.* **121**, e2320687121 (2024).

5. M. Yasuhara, H.-H. M. Huang, M. Reuter, S. Y. Tian, J. D. Cybulski, A. O'Dea, B. L. Mamo, L. J. Cotton, E. di Martino, R. Feng, C. R. Tabor, G. Reygondeau, Q. Zhao, M. T. Warne, K. K. T. Aye, J. Zhang, A. Chao, C.-L. Wei, F. L. Condamine, A. T. Kocsis, W. Kiessling, M. J. Costello, D. P. Tittensor, C. Chaudhary, M. C. Rillo, H. Doi, Y.-W. Dong, T. M. Cronin, E. E. Saupe, H. K. Lotze, K. G. Johnson, W. Renema, J. M. Pandolfi, M. Harzhauser, J. B. C. Jackson, Y. Hong, Hotspots of Cenozoic tropical marine biodiversity. *Oceanogr. Mar. Biol. Ann. Rev.* **60**, 243–300 (2022).

6. W. Renema, D. R. Bellwood, J. C. Braga, K. Bromfield, R. Hall, K. G. Johnson, P. Lunt, C. P. Meyer, L. B. McMonagle, R. J. Morley, A. O'Dea, J. A. Todd, F. P. Wesselingh, M. E. J. Wilson, J. M. Pandolfi, Hopping hotspots: Global shifts in marine biodiversity. *Science* **321**, 654–657 (2008).

7. O. M. Bialik, M. Frank, C. Betzler, R. Zammit, N. D. Waldmann, Two-step closure of the Miocene Indian Ocean Gateway to the Mediterranean. *Sci. Rep.* **9**, 8842 (2019).

8. T. D. Herbert, K. T. Lawrence, A. Tzanova, L. C. Peterson, R. Caballero-Gill, C. S. Kelly, Late Miocene global cooling and the rise of modern ecosystems. *Nat. Geosci.* **9**, 843–847 (2016).

9. K. J. Hsü, W. B. F. Ryan, M. B. Cita, Late Miocene desiccation of the Mediterranean. *Nature* **242**, 240–244 (1973).

10. W. Krijgsman, F. J. Hilgen, I. Raffi, F. J. Sierro, D. S. Wilson, Chronology, causes and progression of the Messinian salinity crisis. *Nature* **400**, 652–655 (1999).

11. R. Flecker, W. Krijgsman, W. Capella, C. de Castro Martíns, E. Dmitrieva, J. P. Mayser, A. Marzocchi, S. Modestu, D. Ochoa, D. Simon, M. Tulbure, B. van den Berg, M. van der Schee, G. de Lange, R. Ellam, R. Govers, M. Gutjahr, F. Hilgen, T. Kouwenhoven, J. Lofi, P. Meijer, F. J. Sierro, N. Bachiri, N. Barhoun, A. C. Alami, B. Chacon, J. A. Flores, J. Gregory, J. Howard, D. Lunt, M. Ochoa, R. Pancost, S. Vincent, M. Z. Yousfi, Evolution of the Late Miocene Mediterranean–Atlantic gateways and their impact on regional and global environmental change. *Earth Sci. Rev.* **150**, 365–392 (2015).

12. W. Krijgsman, W. Capella, D. Simon, F. J. Hilgen, T. J. Kouwenhoven, P. T. Meijer, F. J. Sierro, M. A. Tulbure, B. C. J. van den Berg, M. van der Schee, R. Flecker, The Gibraltar corridor: Watergate of the Messinian salinity crisis. *Mar. Geol.* **403**, 238–246 (2018).

13. I. Vasiliev, V. Karakitsios, I. Bouloubassi, K. Agiadi, G. Kontakiotis, A. Antonarakou, M. Triantaphyllou, A. Gogou, N. Kafousia, M. de Rafélis, S. Zarkogiannis, F. Kaczmar, C. Parinos, N. Pasadakis, Large sea surface temperature, salinity, and productivity-preservation changes preceding the onset of the Messinian Salinity Crisis in the eastern Mediterranean Sea. *Paleoceanogr. Paleoclimatol.* **34**, 182–202 (2019).

14. D. Garcia-Castellanos, A. Villaseñor, Messinian salinity crisis regulated by competing tectonics and erosion at the Gibraltar arc. *Nature* **480**, 359–363 (2011).

15. G. Kontakiotis, G. A. Butiseacă, A. Antonarakou, K. Agiadi, S. D. Zarkogiannis, E. Krsnik, E. Besiou, W. J. Zachariasse, L. Lourens, D. Thivaiou, E. Koskeridou, P. Moissette, A. Mulch, V. Karakitsios, I. Vasiliev, Hypersalinity accompanies tectonic restriction in the eastern Mediterranean prior to the Messinian Salinity Crisis. *Palaeogeogr. Palaeoclimatol. Palaeoecol.* **592**, 110903 (2022).

16. W. Krijgsman, E. J. Rohling, D. V. Palcu, F. Raad, U. Amarathunga, R. Flecker, F. Floringo, A. P. Roberts, F. J. Sierro, G. Aloisi, Causes and consequences of the Messinian salinity crisis. *Nat. Rev. Earth Environ.* **5**, 335–350 (2024).

17. F. Bulian, F. J. Sierro, S. Ledesma, F. J. Jiménez-Espejo, M.-A. Bassetti, Messinian West Alboran Sea record in the proximity of gibraltar: Early signs of Atlantic-Mediterranean gateway restriction. *Mar. Geol.* **434**, 106430 (2021).

18. F. Bulian, T. J. Kouwenhoven, F. J. Jiménez-Espejo, W. Krijgsman, N. Andersen, F. J. Sierro, Impact of the Mediterranean-Atlantic connectivity and the late Miocene carbon shift on deep-sea communities in the Western Alboran Basin. *Palaeogeogr. Palaeoclimatol. Palaeoecol.* **589**, 110841 (2022).

19. M. Sabino, F. Dela Pierre, M. Natalicchio, D. Birgel, S. Gier, J. Peckmann, The response of water column and sedimentary environments to the advent of the Messinian salinity crisis: insights from an onshore deep-water section (Govone, NW Italy). *Geol. Mag.* **158**, 825–841 (2020).

20. W. J. Zachariasse, L. J. Lourens, The Messinian on Gavdos (Greece) and the status of currently used ages for the onset of the MSC and gypsum precipitation. *Newsl. Stratigr.* **55**, 333–360 (2021).

21. M. Natalicchio, F. Dela Pierre, D. Birgel, H. Brumsack, G. Carnevale, R. Gennari, S. Gier, F. Lozar, L. Pellegrino, M. Sabino, B. Schnetger, J. Peckmann, Paleoenvironmental change in a precession-paced succession across the onset of the Messinian salinity crisis: Insight from element geochemistry and molecular fossils. *Palaeogeogr. Palaeoclimatol. Palaeoecol.* **518**, 45–61 (2019).

22. M. Roveri, R. Flecker, W. Krijgsman, J. Lofi, S. Lugli, V. Manzi, F. J. Sierro, A. Bertini, A. Camerlenghi, G. De Lange, R. Govers, F. J. Hilgen, C. Hübscher, P. T. Meijer, M. Stoica, The Messinian Salinity Crisis: Past and future of a great challenge for marine sciences. *Mar. Geol.* **352**, 25–58 (2014).

23. F. Andreetto, G. Aloisi, F. Raad, H. Heida, R. Flecker, K. Agiadi, J. Lofi, S. Blondel, F. Bulian, A. Camerlenghi, A. Caruso, R. Ebner, D. Garcia-Castellanos, V. Gaullier, L. Guibourdenche, Z. Gvirtzman, T. M. Hoyle, P. T. Meijer, J. Moneron, F. J. Sierro, G. Travan, A. Tzevahirtzian, I. Vasiliev, W. Krijgsman, Freshening of the Mediterranean Salt Giant: controversies and certainties around the terminal (Upper Gypsum and Lago-Mare) phases of the Messinian Salinity Crisis. *Earth Sci. Rev.* **216**, 103577 (2021).

24. A. S. Madof, W. B. F. Ryan, C. Bertoni, F. J. Laugier, A. S. Zaki, S. E. Baumgardner, Time-probabilistic approach to the late Miocene Messinian salinity crisis: Implications for a disconnected paratethys. *Terra Nova* **34**, 395–406 (2022).

25. A. Caruso, M.-M. Blanc-Valleron, S. Da Prato, C. Pierre, J. M. Rouchy, The late Messinian “Lago-Mare” event and the Zanclean reflooding in the Mediterranean Sea: New insights from the Cuevas del Almanzora section (Vera Basin, South-Eastern Spain). *Earth Sci. Rev.* **200**, 102993 (2020).

26. D. Garcia-Castellanos, A. Micallef, F. Estrada, A. Camerlenghi, G. Ercilla, R. Periáñez, J. M. Abril, The Zanclean megaflood of the Mediterranean–Searching for independent evidence. *Earth Sci. Rev.* **201**, 103061 (2020).

27. F. D. Por, C. Dimentman, “Continuity of Messinian biota in the Mediterranean Basin” in *Geological Evolution of the Mediterranean Basin* (Springer, 1985), pp. 545–557.

28. C. N. Bianchi, C. Morri, M. Chiantore, M. Montefalcone, V. Parravicini, A. Rovere, “Mediterranean Sea biodiversity between the legacy from the past and a future of change” in *Life in the Mediterranean Sea: A Look at Habitat Changes* (Nova Science Publishers Inc., 2012), pp. 1–55.

29. R. H. Benson, Changes in the ostracods of the Mediterranean with the Messinian salinity crisis. *Palaeogeogr. Palaeoclim. Palaeoecol.* **20**, 147–170 (1976).

30. M. B. Cita, Biodynamic effects of the Messinian salinity crisis on the evolution of planktonic foraminifera in the Mediterranean. *Palaeogeogr. Palaeoclim. Palaeoecol.* **20**, 23–42 (1976).

31. D. Néraudeau, E. Goubert, D. Lacour, J. M. Rouchy, Changing biodiversity of Mediterranean irregular echinoids from the Messinian to the present-day. *Palaeogeogr. Palaeoclimatol. Palaeoecol.* **175**, 43–60 (2001).

32. E. Goubert, D. Néraudeau, J. M. Rouchy, D. Lacour, Foraminiferal record of environmental changes: Messinian of the Los Yesos area (Sorbas Basin, SE Spain). *Palaeogeogr. Palaeoclimatol. Palaeoecol.* **175**, 61–78 (2001).

33. P. Monegatti, S. Raffi, The Messinian marine molluscs record and the dawn of the eastern Atlantic biogeography. *Palaeogeogr. Palaeoclimatol. Palaeoecol.* **297**, 1–11 (2010).

34. K. Agiadi, N. Hohmann, E. Gliozzi, D. Thivaiou, B. Francesca, M. Taviani, G. Bianucci, A. Collareta, L. Londeix, C. Faranda, F. Bulian, E. Koskeridou, F. Lozar, A. M. Mancini, S. Dominici, P. Moissette, I. Bajo Campos, E. Borghi, G. Iliopoulos, A. Antonarakou, G. Kontakiotis, E. Besiou, S. D. Zarkogiannis, M. Harzhauser, F. Sierro, A. Camerlenghi, D. Garcia-Castellanos, Revised marine fossil record of the Mediterranean before and after the Messinian Salinity Crisis, v3, Zenodo (2024); https://doi.org/10.5281/zenodo.12698765.

35. K. Agiadi, N. Hohmann, E. Gliozzi, D. Thivaiou, B. Francesca, M. Taviani, G. Bianucci, A. Collareta, L. Londeix, C. Faranda, F. Bulian, E. Koskeridou, F. Lozar, A. M. Mancini, S. Dominici, P. Moissette, I. B. Campos, E. Borghi, G. Iliopoulos, A. Antonarakou, G. Kontakiotis, E. Besiou, S. D. Zarkogiannis, M. Harzhauser, F. Sierro, A. Camerlenghi, D. Garcia-Castellanos, A revised marine fossil record of the Mediterranean before and after the Messinian Salinity Crisis. *Earth Sys. Sci. Data* , 1–11 (2024).

36. W. Capella, N. Barhoun, R. Flecker, F. J. Hilgen, T. Kouwenhoven, L. C. Matenco, F. J. Sierro, M. A. Tulbure, M. Z. Yousfi, W. Krijgsman, Palaeogeographic evolution of the late Miocene Rifian Corridor (Morocco): Reconstructions from surface and subsurface data. *Earth Sci. Rev.* **180**, 37–59 (2018).

37. G. Carnevale, R. Gennari, F. Lozar, M. Natalicchio, L. Pellegrino, F. Dela Pierre, Living in a deep desiccated Mediterranean Sea: An overview of the Italian fossil record of the Messinian salinity crisis. *Boll. Soc. Paleontol. Ital.* **58**, 109–140 (2019).

38. G. Carnevale, W. Schwarzhans, Marine life in the Mediterranean during the Messinian Salinity Crisis: A paleoichthyological perspective. *Riv. Ital. Paleontol. Stratigr.* **128**, 283–324 (2022).

39. A. Baselga, Partitioning the turnover and nestedness components of beta diversity. *Glob. Ecol. Biogeogr.* **19**, 134–143 (2010).

40. A. Vertino, J. Stolarski, F. R. Bosellini, M. Taviani, "Mediterranean corals through time: From Miocene to Present" in *The Mediterranean Sea*, S. Goffredo, Z. Dubinsky, Eds. (Springer, 2014), pp. 257–274.

41. R. H. Benson, The origin of the psychrosphere as recorded in changes of deep-sea ostracode assemblages. *Lethaia* **8**, 69–83 (1975).

42. D. J. Ehret, B. J. Macfadden, D. S. Jones, T. J. Devries, D. A. Foster, R. Salas-Gismondi, Origin of the white shark *Carcharodon* (Lamniformes: Lamnidae) based on recalibration of the Upper Neogene Pisco Formation of Peru. *Palaeontology* **55**, 1139–1153 (2012).

43. A. Collareta, S. Casati, A. Di Cencio, G. Bianucci, Quaternary records of *Carcharodon carcharias* (LINNAEUS, 1758) in Tuscany (central Italy): Implications for the palaeobiology of Mediterranean white sharks. *Neues Jahrb. Geol. Paläontol.* **309**, 65–76 (2023).

44. F. R. Bosellini, C. Perrin, Estimating Mediterranean Oligocene–Miocene sea-surface temperatures: An approach based on coral taxonomic richness. *Palaeogeogr. Palaeoclimatol. Palaeoecol.* **258**, 71–88 (2008).

45. S. Y. Tian, M. Yasuhara, F. L. Condamine, H.-H. M. Huang, A. G. S. Fernando, Y. M. Aguilar, H. Pandita, T. Irizuki, H. Iwatani, C. P. Shin, W. Renema, T. Kase, Cenozoic history of the tropical marine biodiversity hotspot. *Nature* **632**, 343–349 (2024).

46. C. Perrin, F. R. Bosellini, Paleobiogeography of scleractinian reef corals: Changing patterns during the Oligocene–Miocene climatic transition in the Mediterranean. *Earth-Sci. Rev.* **111**, 1–24 (2012).

47. J. P. Chevalier, Recherches sur les madréporaires et les formations récifales Miocènes de la Méditerranée occidentale. *Mem. Soc. Geol. France* **93**, 562 (1962).

48. B. Sabelli, M. Taviani, The paleobiogeographic distribution of the Mediterranean benthic mollusks and the Messinian salinity crisis or where did the mollusks go? *Ann. Géologie Pays Hell.* **32**, 263–269 (1984).

49. R. Gennari, V. Manzi, L. Angeletti, A. Bertini, U. Biffi, A. Ceregato, C. Faranda, E. Gliozzi, S. Lugli, E. Menichetti, A. Rosso, M. Roveri, M. Taviani, A shallow water record of the onset of the Messinian salinity crisis in the adriatic foredeep (Legnagnone section, Northern Apennines). *Palaeogeogr. Palaeoclim. Palaeoecol.* **386**, 145–164 (2013).

50. D. Néraudeau, S. Barbe, D. Mercier, J. Roman, Signatures paléoclimatiques des échinides du Néogène final atlantique à faciès redonien. *Ann. Paléontol.* **89**, 153–170 (2003).

51. G. Booth-Rea, C. R. Ranero, I. Grevemeyer, The Alboran volcanic-arc modulated the Messinian faunal exchange and salinity crisis. *Sci. Rep.* **8**, 13015 (2018).

52. H. Heida, D. Garcia-Castellanos, I. Jimenez-Munt, F. Estrada, G. Ercilla, D. Do Couto, A. Ammar, Seaway restriction, sea level drop and erosion in the Alboran Basin from a paleotopographic reconstruction for the Messinian Salinity Crisis. *Mar. Geol.* **474**, 107300 (2024).

53. A. M. Mancini, R. Gennari, P. Ziveri, P. G. Mortyn, D. J. Stolwijk, F. Lozar, Calcareous nannofossil and foraminiferal trace element records in the Sorbas Basin: A new piece of the Messinian Salinity Crisis onset puzzle. *Palaeogeogr. Palaeoclimatol. Palaeoecol.* **554**, 109796 (2020).

54. R. Gladstone, R. Flecker, P. Valdes, D. Lunt, P. Markwick, The Mediterranean hydrologic budget from a Late Miocene global climate simulation. *Palaeogeogr. Palaeoclimatol. Palaeoecol.* **251**, 254–267 (2007).

55. T. Kouwenhoven, C. Morigi, A. Negri, S. Giunta, W. Krijgsman, J.-M. Rouchy, Paleoenvironmental evolution of the eastern Mediterranean during the Messinian: Constraints from integrated microfossil data of the Pissouri Basin (Cyprus). *Mar. Micropaleontol.* **60**, 17–44 (2006).

56. R. H. Benson, K. R.-E. Bied, G. Bonaduce, An important current reversal (influx) in the Rifian Corridor (Morocco) at the Tortonian-Messinian boundary: The end of Tethys Ocean. *Paleoceanography* **6**, 165–192 (1991).

57. G. Bianucci, G. Carone, D. P. Domning, W. Landini, L. Rook, S. Sorbi, Peri-Messinian dwarfing in Mediterranean *Metaxytherium* (Mammalia: Sirenia): Evidence of habitat degradation related to the Messinian salinity crisis. *Garyounis Sci. Bull.* **5**, 145–157 (2008).

58. M. T. Clementz, S. Sorbi, D. P. Domning, Evidence of Cenozoic environmental and ecological change from stable isotope analysis of sirenian remains from the Tethys-Mediterranean region. *Geology* **37**, 307–310 (2009).

59. C. G. C. van Baak, M. Stoica, A. Grothe, E. Aliyeva, W. Krijgsman, Mediterranean-Paratethys connectivity during the Messinian salinity crisis: The Pontian of Azerbaijan. *Glob. Planet. Change* **141**, 63–81 (2016).

60. D. Barra, G. Bonaduce, F. Sgarella, Paleoenvironmental bottom water conditions in the early Zanclean of the Capo Rosselo area (Agrigento, Sicily). *Boll. Soc. Paleontol. Ital.* **37**, 61–98 (1998).

61. A. L. Stigall, J. E. Bauer, A. R. Lam, D. F. Wright, Biotic immigration events, speciation, and the accumulation of biodiversity in the fossil record. *Glob. Planet. Change* **148**, 242–257 (2017).

62. G. Bianucci, M. Gatt, R. Catanzariti, S. Sorbi, C. G. Bonavia, R. Curmi, A. Varola, Systematics, biostratigraphy and evolutionary pattern of the Oligo-Miocene marine mammals from the Maltese Islands. *Geobios* **44**, 549–585 (2011).

63. C. M. Peredo, M. D. Uhen, Exploration of marine mammal paleogeography in the Northern Hemisphere over the Cenozoic using beta diversity. *Palaeogeogr. Palaeoclimatol. Palaeoecol.* **449**, 227–235 (2016).

64. M. D. Uhen, N. D. Pyenson, Diversity estimates, biases, and historiographic effects: Resolving cetacean diversity in the Tertiary. *Palaeontol. Electron.* **10**, 22 (2007).

65. S. Dominici, M. Benvenuti, M. Forli, C. Bogi, A. Guerrini, Upper Miocene molluscs of Monti Livornesi (Tuscany, Italy): Biotic changes across environmental gradients. *Palaeogeogr. Palaeoclimatol. Palaeoecol.* **527**, 103–117 (2019).

66. S. Dominici, M. Forli, Lower Pliocene molluscs from southern Tuscany (Italy). *Boll. Soc. Paleontol. Ital.* **60**, 69–98 (2021).

67. D. M. Raup, Species diversity in the phanerozoic: An interpretation. *Paleobiology* **2**, 289–297 (1976).

68. J. B. C. Jackson, K. G. Johnson, Measuring past biodiversity. *Science* **293**, 2401–2404 (2001).

69. S. Dominici, M. Forli, C. Bogi, A. Guerrini, M. Benvenuti, Paleobiology from museum collections: Comparing historical and novel data on upper Miocene molluscs of the Livorno Hills. *Riv. Ital. Paleontol. Stratigr.* **126**, 65–109 (2020).

70. S. Dominici, S. Danise, M. Benvenuti, Pliocene stratigraphic paleobiology in Tuscany and the fossil record of marine megafauna. *Earth-Sci. Rev.* **176**, 277–310 (2018).

71. E. Koskeridou, C. Giamali, A. Antonarakou, G. Kontakiotis, V. Karakitsios, Early Pliocene gastropod assemblages from the eastern Mediterranean (SW Peloponnese, Greece) and their palaeobiogeographic implications. *Geobios* **50**, 267–277 (2017).

72. A. C. Allwood, I. W. Burch, J. M. Rouchy, M. Coleman, Morphological biosignatures in gypsum: Diverse formation processes of Messinian (~6.0 Ma) gypsum stromatolites. *Astrobiology* **13**, 870–886 (2013).

73. M. Natalicchio, D. Birgel, J. Peckmann, F. Lozar, G. Carnevale, X. Liu, K.-U. Hinrichs, F. Dela Pierre, An archaeal biomarker record of paleoenvironmental change across the onset of the Messinian salinity crisis in the absence of evaporites (Piedmont Basin, Italy). *Org. Geochem.* **113**, 242–253 (2017).

74. A. S. Madof, C. Bertoni, J. Lofi, Discovery of vast fluvial deposits provides evidence for drawdown during the late Miocene Messinian salinity crisis. *Geology* **47**, 171–174 (2019).

75. A. Meilijson, J. Steinberg, F. Hilgen, O. M. Bialik, N. D. Waldmann, Y. Makovsky, Deep-basin evidence resolves a 50-year-old debate and demonstrates synchronous onset of Messinian evaporite deposition in a non-desiccated Mediterranean. *Geology* **46**, 243–246 (2018).

76. M. W. Cadotte, K. Carscadden, N. Mirotchnick, Beyond species: Functional diversity and the maintenance of ecological processes and services. *J. Appl. Ecol.* **48**, 1079–1087 (2011).

77. C. Amadori, D. Garcia-Castellanos, G. Toscani, P. Sternai, R. Fantoni, M. Ghielmi, A. D. Giulio, Restored topography of the Po Plain-Northern Adriatic region during the Messinian base-level drop—Implications for the physiography and compartmentalization of the palaeo-Mediterranean basin. *Basin Res.* **30**, 1247–1263 (2018).

78. F. F. Steininger, F. Rögl, Paleogeography and palinspastic reconstruction of the Neogene of the Mediterranean and Paratethys. *Geol. Soc. Lond. Spec. Publ.* **17**, 659–668 (1984).

79. I. Cornacchia, M. Brandano, S. Agostini, Miocene paleoceanographic evolution of the Mediterranean area and carbonate production changes: A review. *Earth Sci. Rev.* **221**, 103785 (2021).

80. R. W. H. Butler, W. H. Lickorish, M. Grasso, H. M. Pedley, L. Ramberti, Tectonics and sequence stratigraphy in Messinian basins, Sicily: Constraints on the initiation and termination of the Mediterranean salinity crisis. *Geol. Soc. Am. Bull.* **107**, 425–439 (1995).

81. L. Caracciolo, P. Gramigna, S. Critelli, A. B. Calzona, F. Russo, Petrostratigraphic analysis of a Late Miocene mixed siliciclastic–carbonate depositional system (Calabria, Southern Italy): Implications for Mediterranean paleogeography. *Sediment. Geol.* **284–285**, 117–132 (2013).

82. M. Henriquet, S. Dominguez, G. Barreca, J. Malavieille, C. Monaco, Structural and tectono-stratigraphic review of the Sicilian orogen and new insights from analogue modeling. *Earth-Sci. Rev.* **208**, 103257 (2020).

83. A. Micallef, A. Camerlenghi, A. Georgiopoulou, D. Garcia-Castellanos, M.-A. Gutscher, C. Lo Iacono, V. A. I. Huvenne, J. J. Mountjoy, C. K. Paull, T. Le Bas, D. Spatola, L. Facchin,

D. Accettella, Geomorphic evolution of the Malta Escarpment and implications for the Messinian evaporative drawdown in the eastern Mediterranean Sea. *Geomorphology* **327**, 264–283 (2019).

84. F. Bache, S.-M. Popescu, M. Rabineau, C. Gorini, J.-P. Suc, G. Clauzon, J.-L. Olivet, J.-L. Rubino, M. C. Melinte-Dobrinescu, F. Estrada, L. Londeix, R. Armijo, B. Meyer, L. Jolivet, G. Jouannic, E. Leroux, D. Aslanian, A. T. D. Reis, L. Mocochain, N. Dumurdžanov, I. Zagorchev, V. Lesić, D. Tomić, M. N. Çağatay, J.-P. Brun, D. Sokoutis, I. Csato, G. Ucarkus, Z. Çakir, A two-step process for the reflooding of the Mediterranean after the Messinian Salinity Crisis. *Basin Res.* **24**, 125–153 (2012).

85. R. J. Whittaker, K. J. Willis, R. Field, Scale and species richness: Towards a general, hierarchical theory of species diversity. *J. Biogeogr.* **28**, 453–470 (2001).

86. P. Koleff, K. J. Gaston, J. J. Lennon, Measuring beta diversity for presence–absence data. *J. Anim. Ecol.* **72**, 367–382 (2003).

87. W. Ulrich, M. Almeida-Neto, On the meanings of nestedness: Back to the basics. *Ecography* **35**, 865–871 (2012).

88. P. H. Raven, R. E. Gereau, P. B. Phillipson, C. Chatelain, C. N. Jenkins, C. Ulloa Ulloa, The distribution of biodiversity richness in the tropics. *Sci. Adv.* **6**, eabc6228 (2020).

89. N. Hohmann, K. Agiadi, Supplementary code for “The marine biodiversity impact of the Late Miocene Mediterranean salinity crisis,” v1.2.0. Zenodo (2024); https://doi.org/10.5281/zenodo.12678336.

90. P. S. Barton, S. A. Cunningham, A. D. Manning, H. Gibb, D. B. Lindenmayer, R. K. Didham, The spatial scaling of beta diversity. *Glob. Ecol. Biogeogr.* **22**, 639–647 (2013).

91. V. J. Roden, Á. T. Kocsis, M. Zuschin, W. Kiessling, Reliable estimates of beta diversity with incomplete sampling. *Ecology* **99**, 1051–1062 (2018).

92. L. B. Ilyina, I. G. Shcherba, S. Khondkarian, “Map 8: Middle late Miocene (late Tortonian–early Messinian–early Maeotian–late Pannonian)” in *Lithological-Paleogeographic Maps of*

Paratethys 10 Maps Late Eocene to Pliocene, S. V. Popov, F. Rogl, A. Y. Rozanov, F. F. Steininger, I. G. Shcherba, M. Kovac, Eds. (Courier Forschungsinstitut Senckenberg, 2004), pp. 31–34.

93. J. C. Braga, J. M. Martín, R. Riding, J. Aguirre, I. M. Sánchez-Almazo, J. Dinarès-Turell, Testing models for the Messinian salinity crisis: The Messinian record in Almería, SE Spain. *Sediment. Geol.* **188–189**, 131–154 (2006).

94. J. M. Martin, J. C. Braga, I. Sánchez-Almazo, The Messinian record of the outcropping marginal Alboran basin deposits: Significance and implications. *Proc. Ocean Drill. Program Sci. Results* **161**, 543–551 (1999).

95. A. Tzanova, T. D. Herbert, L. Peterson, Cooling Mediterranean Sea surface temperatures during the Late Miocene provide a climate context for evolutionary transitions in Africa and Eurasia. *Earth Planet. Sci. Lett.* **419**, 71–80 (2015).

96. F. Pilade, I. Vasiliev, D. Birgel, F. Della Pierre, M. Natalicchio, A. Mancini, G. Carneavale, R. Gennari, Deciphering the termination of the Messinian salinity crisis: The alkenone record of the Miocene–Pliocene transition in the northern Mediterranean. *Palaeogeogr. Palaeoclim. Palaeoecol.* **631**, 111831 (2023).

97. J. Jaminski, The mid-Cretaceous palaeoenvironmental conditions in the Polish Carpathians—A palynological approach. *Rev. Palaeobot. Palynol.* **87**, 43–50 (1995).

98. G.-J. Reichart, H. Brinkhuis, Late Quaternary Protoperidinium cysts as indicators of paleoproductivity in the northern Arabian Sea. *Mar. Micropaleontol.* **49**, 303–315 (2003).

99. J.-L. Turon, "Le palynoplancton dans l'environnement actuel de l'Atlantique nord-oriental: Évolution climatique et hydrologique depuis le dernier maximum glaciaire," thesis, Université de Bordeaux I (1984).

100. K. A. F. Zonneveld, G. J. M. Versteegh, G. J. de Lange, Preservation of organic-walled dinoflagellate cysts in different oxygen regimes: A 10,000 year natural experiment. *Mar. Micropaleontol.* **29**, 393–405 (1997).

101. B. C. J. van den Berg, F. J. Sierro, F. J. Hilgen, R. Flecker, J. C. Larrasoaña, W. Krijgsman, J. A. Flores, M. P. Mata, E. Bellido Martín, J. Civis, J. A. González-Delgado, Astronomical tuning for the upper Messinian Spanish Atlantic margin: Disentangling basin evolution, climate cyclicity and MOW. *Glob. Planet. Change* **135**, 89–103 (2015).

102. M.-M. Blanc-Valleron, C. Pierre, J. P. Caulet, A. Caruso, J.-M. Rouchy, G. Cespuglio, R. Sprovieri, S. Pestrea, E. Di Stefano, Sedimentary, stable isotope and micropaleontological records of paleoceanographic change in the Messinian Tripoli Formation (Sicily, Italy). *Palaeogeogr. Palaeoclimatol. Palaeoecol.* **185**, 255–286 (2002).

103. F. J. Sierro, J. A. Flores, G. Francés, A. Vazquez, R. Utrilla, I. Zamarreño, H. Erlenkeuser, M. A. Barcena, Orbitally-controlled oscillations in planktic communities and cyclic changes in western Mediterranean hydrography during the Messinian. *Palaeogeogr. Palaeoclimatol. Palaeoecol.* **190**, 289–316 (2003).

104. R. Selli, Il Messiniano Mayer Eymar 1867, proposta di un neostratotipo. *G. Geol.* **28**, 1–33 (1960).

105. F. Sierro, J. Flores, J. Civis, J. Delgado, G. Frances, Late Miocene globorotaliid event-stratigraphy and biogeography in the NE-Atlantic and Mediterranean. *Mar. Micropaleontol.* **21**, 143–167 (1993).

106. R. Gennari, F. Lozar, E. Turco, F. Dela Pierre, S. Lugli, V. Manzi, M. Natalicchio, M. Roveri, B. C. Schreiber, M. Taviani, Integrated stratigraphy and paleoceanographic evolution of the pre-evaporitic phase of the Messinian salinity crisis in the Eastern Mediterranean as recorded in the Tokhni section (Cyprus island). *Newsl. Stratigr.* **51**, 33–55 (2018).

107. V. Karakitsios, M. Roveri, S. Lugli, V. Manzi, R. Gennari, A. Antonarakou, M. Triantaphyllou, K. Agiadi, G. Kontakiotis, N. Kafousia, M. de Rafelis, A record of the Messinian salinity crisis in the eastern Ionian tectonically active domain (Greece, eastern Mediterranean). *Basin Res.* **29**, 203–233 (2017).

108. J. Bijma, W. W. Faber, C. Hemleben, Temperature and salinity limits for growth and survival of some planktonic foraminifers in laboratory cultures. *J. Foraminifer. Res.* **20**, 95–116 (1990).

109. H. M. van de Poel, Foraminiferal biostratigraphy and palaeoenvironments of the Miocene-Pliocene carboneras-nijar basin (SE Spain). *Scr. Geol.* **102**, 1–32 (1992).

110. K. Kaiho, Benthic foraminiferal dissolved-oxygen index and dissolved-oxygen levels in the modern ocean. *Geology* **22**, 719–722 (1994).

111. T. J. Kouwenhoven, M.-S. Seidenkrantz, G. J. van der Zwaan, Deep-water changes: The near-synchronous disappearance of a group of benthic foraminifera from the Late Miocene Mediterranean. *Palaeogeogr. Palaeoclimatol. Palaeoecol.* **152**, 259–281 (1999).

112. T. J. Kouwenhoven, F. J. Hilgen, G. J. van der Zwaan, Late Tortonian–early Messinian stepwise disruption of the Mediterranean–Atlantic connections: Constraints from benthic foraminiferal and geochemical data. *Palaeogeogr. Palaeoclimatol. Palaeoecol.* **198**, 303–319 (2003).

113. M.-S. Seidenkrantz, T. J. Kouwenhoven, F. J. Jorissen, N. J. Shackleton, G. J. van der Zwaan, Benthic foraminifera as indicators of changing Mediterranean–Atlantic water exchange in the late Miocene. *Mar. Geol.* **163**, 387–407 (2000).

114. F. J. Sierro, J. A. Flores, I. Zamarreño, A. Vázquez, R. Utrilla, G. Francés, F. J. Hilgen, W. Krijgsman, Messinian pre-evaporite sapropels and precession-induced oscillations in western Mediterranean climate. *Mar. Geol.* **153**, 137–146 (1999).

115. K. Agiadi, I. Vasiliev, G. A. Butiseacă, G. Kontakiotis, D. Thivaiou, E. Besiou, S. D. Zarkogiannis, E. Koskeridou, A. Antonarakou, A. Mulch, Coupled otolith and foraminifera oxygen and carbon stable isotopes evidence paleoceanographic changes and fish metabolic responses. *EGUsphere* **2024**, 1–18 (2024).

116. F. Bulian, T. J. Kouwenhoven, N. Andersen, W. Krijgsman, F. J. Sierro, Reflooding and repopulation of the Mediterranean Sea after the Messinian salinity crisis: Benthic

foraminifer assemblages and stable isotopes of Spanish basins. *Mar. Micropaleontol.* **176**, 102160 (2022).

117. S. M. Iaccarino, M. B. Cita, S. Gaboardi, G. M. Gruppini, 15. High-resolution biostratigraphy at the Miocene/Pliocene boundary in holes 974B and 975B, Western Mediterranean. *Proc. Ocean Drill. Program Sci. Results* **161**, 197–221 (1999).

118. S. Spezzaferri, M. B. Cita, J. A. McKenzie, The Miocene/Pliocene boundary in the eastern Mediterranean: Results from sites 967 and 969. *Proc. Ocean Drill. Program Sci. Results* **160**, 9–28 (1998).

119. I. Mazzini, G. Aiello, P. Frenzel, A. Pint, Marine and marginal marine Ostracods as proxies in geoarcheology. *Mar. Micropaleontol.* **174**, 102054 (2022).

120. M. Marchegiano, A. Francke, E. Gliozzi, B. Wagner, D. Ariztegui, High-resolution palaeohydrological reconstruction of central Italy during the Holocene. *Holocene* **29**, 481–492 (2019).

121. F. Grossi, E. Gliozzi, P. Anadon, F. Castorina, M. Voltaggio, Is *Cyprideis agrigentina* Decima a good paleosalinometer for the Messinian Salinity Crisis? Morphometrical and geochemical analyses from the Eraclea Minoa section (Sicily). *Palaeogeogr. Palaeoclim. Palaeoecol.* **419**, 75–89 (2015).

122. B. Bechor, S. Avnaim-Katav, S. Mischke, S. Miko, O. Hasan, M. Grisonic, I. R. Rossi, B. Herut, N. Taha, N. Porat, D. Sivan, How can past sea level be evaluated from traces of anthropogenic layers in ancient saltpans? *PLOS ONE* **18**, e0287977 (2023).

123. N. V. Aladin, "Salinity tolerance, morphology and physiology of the osmoregulation organs in Ostracoda with special reference to Ostracoda from the Aral Sea" in *Ostracoda in the Earth and Life Sciences*, P. Jones, K. McKenzie, Eds. (A.A. Balkema, 1993), pp. 387–404.

124. D. Keyser, N. Aladin, Noding in *Cyprideis torosa* and its causes. *Stud. Quat.* **21**, 19–24 (2004).

125. P. Frenzel, I. Schulze, A. Pint, Noding of Cyprideis torosa valves (Ostracoda) – A proxy for salinity? New data from field observations and a long-term microcosm experiment. *Int. Rev. Hydrobiol.* **97**, 314–329 (2012).

126. A. Pint, P. Frenzel, R. Fuhrmann, B. Scharf, V. Wennrich, Distribution of *Cyprideis torosa* (Ostracoda) in quaternary athalassic sediments in Germany and its application for palaeoecological reconstructions. *Int. Rev. Hydrobiol.* **97**, 330–355 (2012).

127. I. Boomer, P. Frenzel, M. Feike, Salinity-driven size variability in *Cyprideis torosa* (Ostracoda, Crustacea). *J. Micropalaeontol.* **36**, 63–69 (2017).

128. S. Iaccarino, A. Bossio, Paleoenvironment of the uppermost Messinian sequences in the western Mediterranean (Sites 974, 975, and 978). *Proc. Ocean Drill. Program Sci. Results* **161**, 529–541 (1999).

129. R. H. Benson, "35. The paleoecology of the ostracods of DSDP Leg 42A" (Initial Reports, vol. 42, Deep Sea Drilling Project and Publications, 1978), pp. 777–787.

130. D. Cosentino, V. Bracone, C. D'Amico, P. Cipollari, D. Esu, C. Faranda, V. Frezza, E. Gliozzi, F. Grossi, P. Guerrieri, A. Iadanza, T. Kotsakis, I. Soulié-Märsche, The record of the Messinian salinity crisis in mobile belts: Insights from the Molise allochthronous units (southern Apennines, Italy). *Palaeogeogr. Palaeoclim. Palaeoecol.* **503**, 112–130 (2018).

131. G. Bonaduce, F. Sgarrella, Paleoecological interpretation of the latest Messinian sediments from southern Sicily (Italy). *Soc. Geol. Ital. Mem.* **54**, 83–91 (1999).

132. P. Moissette, S. Pouyet, Bryozoan faunas and the Messinian salinity crisis. *Ann. Inst. Geol. Publ. Hung.* **70**, 447–453 (1987).

133. P. Moissette, Changes in bryozoan assemblages and bathymetric variations examples from the Messinian of northwest Algeria. *Palaeogeogr. Palaeoclimatol. Palaeoecol.* **155**, 305–326 (2000).

134. P. Moissette, A. Antonarakou, G. Kontakiotis, J.-J. Cornée, V. Karakitsios, Bryozoan faunas at the Tortonian-Messinian transition. A palaeoenvironmental case study from Crete Island, eastern Mediterranean. *Geodiversitas* **43**, 1365–1400 (2021).

135. J. Roman, Echinodermes actuels et fossiles. Actes du VI° Séminaire international sur les échinodermes, 19–22 septembre 1988. *Vie Mar Hors Ser.* **10**, 39–48 (1989).

136. H. B. Fell, "The ecology of ophiuroids" in *Physiology of Echinodermata*, R. A. Boolootian, Ed. (Interscience Publishers, 1966), pp. 129–143.

137. I. A. Santos, G. C. Castellano, C. A. Freire, Direct relationship between osmotic and ionic conforming behavior and tissue water regulatory capacity in echinoids. *Comp. Biochem. Physiol. A Mol. Integr. Physiol.* **164**, 466–476 (2013).

138. L. Sorbini, R. Tirapelle Rancan, Messinian fossil fish of the Mediterranean. *Palaeogeogr. Palaeoclimatol. Palaeoecol.* **29**, 143–154 (1979).

139. K. Agiadi, A. Antonarakou, G. Kontakiotis, N. Kafousia, P. Moissette, J.-J. Cornée, E. Manoutsoglou, V. Karakitsios, Connectivity controls on the late Miocene eastern Mediterranean fish fauna. *Int. J. Earth Sci.* **106**, 1147–1159 (2017).

140. W. Schwarzhans, K. Agiadi, G. Carnevale, Late Miocene–Early Pliocene evolution of Mediterranean gobies and their environmental and biogeographic significance. *Riv. Ital. Paleontol. Stratigr.* **126**, 657–724 (2020).

141. C.-H. Lin, R. Brzobohatý, D. Nolf, A. Girone, Tortonian teleost otoliths from northern Italy: Taxonomic synthesis and stratigraphic significance. *Eur. J. Taxon.* **322**, 1–44 (2017).

142. D. Nolf, E. Steurbaut, Révision des otolithes de téléostéens du Tortonien stratotypique et de Montegibbio (Miocène Supérieur de l'Italie septentrionale). *Meded. Werkgr. Tert. Kwart. Geol.* **20**, 143–197 (1983).

143. A. Girone, D. Nolf, O. Cavallo, Fish otoliths from the pre-evaporitic (Early Messinian) sediments of northern Italy: Their stratigraphic and palaeobiogeographic significance. *Facies* **56**, 399–432 (2010).

144. W. Landini, L. Sorbini, Données récentes sur les téléostéens du Miocène et du Pliocène d'Italie. *Geobios* **25**, 151–157 (1992).

145. K. Agiadi, C. Giamali, A. Girone, P. Moissette, E. Koskeridou, V. Karakitsios, The Zanclean marine fish fauna and palaeoenvironmental reconstruction of a coastal marine setting in the eastern Mediterranean. *Paleobiodivers. Paleoenviron.* **100**, 773–792 (2020).

146. D. Nolf, A. Girone, Otolithes de poissons du Pliocène inferieur (Zancleen) des environs d'Alba (Piemont) et de la cote ligure. *Riv. Piemontese Storia Nat.* **27**, 77–114 (2006).

147. C. Arambourg, Les poissons fossiles d'Oran. *Matér. Pour Carte Géologique L'Algérie* **6**, 1–218 (1927).

148. E. Menesini, Ittiodontoliti miocenici di terra d'Otranto. *Palaeontol. Ital.* **65**, 1–61 (1969).

149. E. Menesini, I Pesci miocenici delle Arenarie di Ponsano. *Atti Della Soc. Toscana Sci. Nat. Memorie Ser. A.* **74**, 1–22 (1967).

150. T. Pawellek, S. Adnet, H. Cappetta, E. Métais, M. Salem, M. Brunet, J.-J. Jaeger, Discovery of an earliest Pliocene relic tropical fish fauna in a newly detected cliff section (Sabratah Basin, NW Libya). *N. Jb. Geol. Paläont.* **266**, 93–114 (2012).

151. A. Collareta, M. Merella, F. H. Mollen, S. Casati, A. Di Cencio, The extinct catshark *Pachyscyllium distans* (Probst, 1879) (Elasmobranchii: Carcharhiniformes) in the Pliocene of the Mediterranean Sea. *N. Jb. Geol. Paläont.* **295**, 129–139 (2020).

152. A. Collareta, S. Casati, A. Di Cencio, G. Bianucci, The deep past of the white shark, *Carcharodon carcharias*, in the Mediterranean Sea: A synthesis of its palaeobiology and palaeoecology. *Life* **13**, 2085 (2023).

153. S. Marsili, Revision of the teeth of the genus *Carcharhinus* (Elasmobranchii; Carcharhinidae) from the Pliocene of Tuscany, Italy. *Riv. Ital. Paleontol. Stratigr.* **113**, 79–95 (2007).

154. W. Landini, A. Collareta, G. Bianucci, The origin of biogeographic segregation in the copper shark (*Carcharhinus brachyurus*): An integrative reconstruction based on neontological and paleontological data. *Vie Milieu* **70**, 117–132 (2020).

155. T. Reinecke, S. Louwye, U. Havekost, H. Moths, The elasmobranch fauna of the late burdigalian, Miocene, at Werder-Uesen, Lower Saxony, Germany, and its relationships with early Miocene faunas in the North Atlantic Central Paratethys and Mediterranean. *Palaeontos* **20**, 1–170 (2011).

156. H. Cappetta, D. Nolf, Les sélaciens du pliocène inférieur de Le-Puget-Sur-Argens (Sud-Est de la France). *Palaeontogr. Abt. A.* **218**, 49–67 (1991).

157. L. Dewaele, O. Lambert, S. Louwye, A critical revision of the fossil record, stratigraphy and diversity of the Neogene seal genus *Monotherium* (Carnivora, Phocidae). *R. Soc. Open Sci.* **5**, 171669 (2018).

158. C. De Muizon, Premier signalement de monachinae (Phocidae: Mammalia) dans le sahèlien (Miocène Supérieue) d'Oran (Algérie). *Palaeovertebrata* **11**, 181–194 (1981).

159. A. A. Karamanlidis, P. Dendrinos, P. F. de Larrinoa, A. C. Gücü, W. M. Johnson, C. O. Kiraç, R. Pires, The Mediterranean monk seal *Monachus monachus*: Status, biology, threats, and conservation priorities. *Mammal Rev.* **46**, 92–105 (2016).

160. D. Kahle, H. Wickham, ggmap: Spatial visualization with ggplot2. *R J.* **5**, 144–161 (2013).
